# Supplementary figures and images for: Variation in anthelmintic responses are driven by genetic differences among diverse C. elegans wild strains
Source: PLoS Pathog. 2023 Apr 3;19(4):e1011285. doi: 10.1371/journal.ppat.1011285 (PMC10101645; doi:10.1371/journal.ppat.1011285)

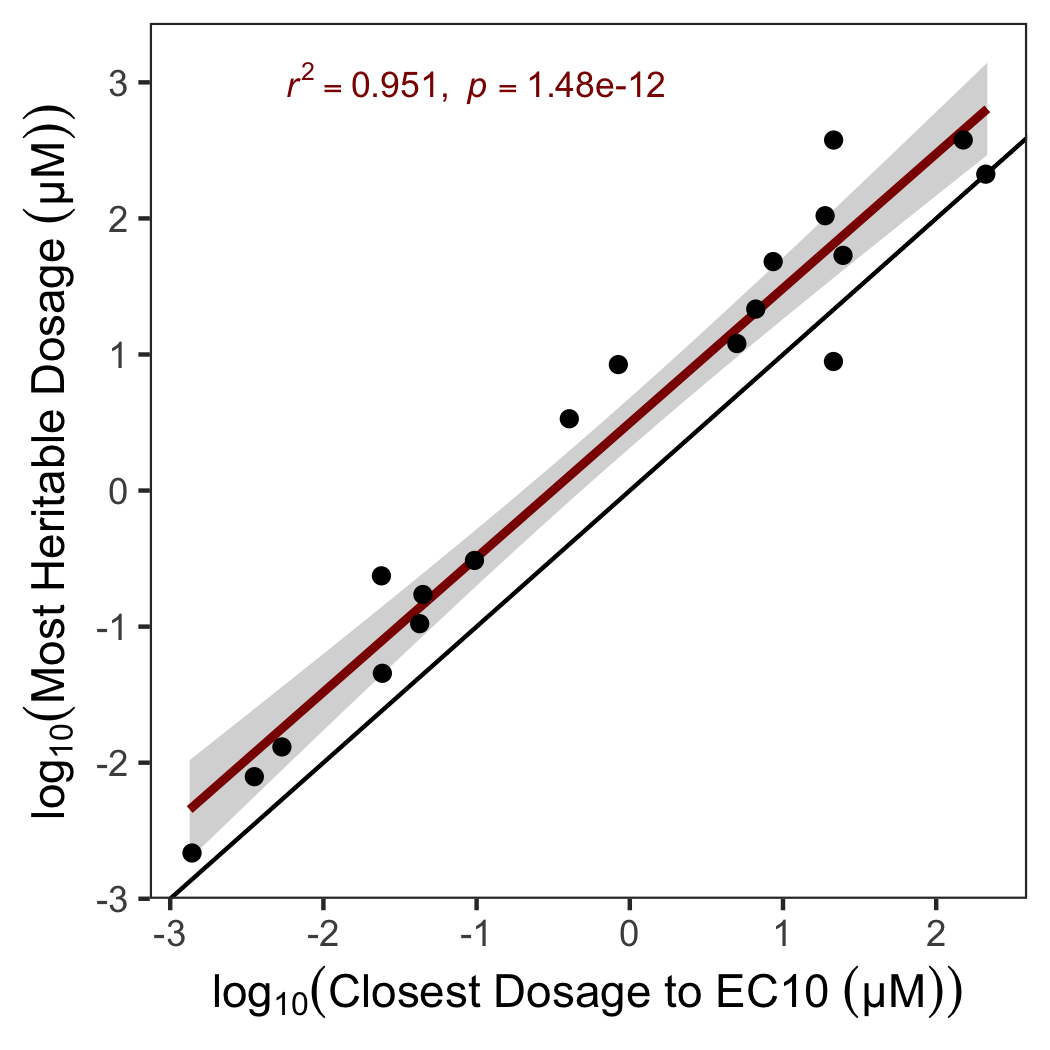

Supplement: S1 Fig — The log-transformed exposure that elicited the most heritable response to each anthelmintic (y-axis) is plotted against the log-transformed exposure of that same anthelmintic nearest to the inferred EC10 from the dose-response assessment. The exposure closest to the EC10 across all anthelmintics exhibited significant explanatory power to determine the exposure that elicited heritable phenotypic variation. (TIF) [file ppat.1011285.s007.tif]

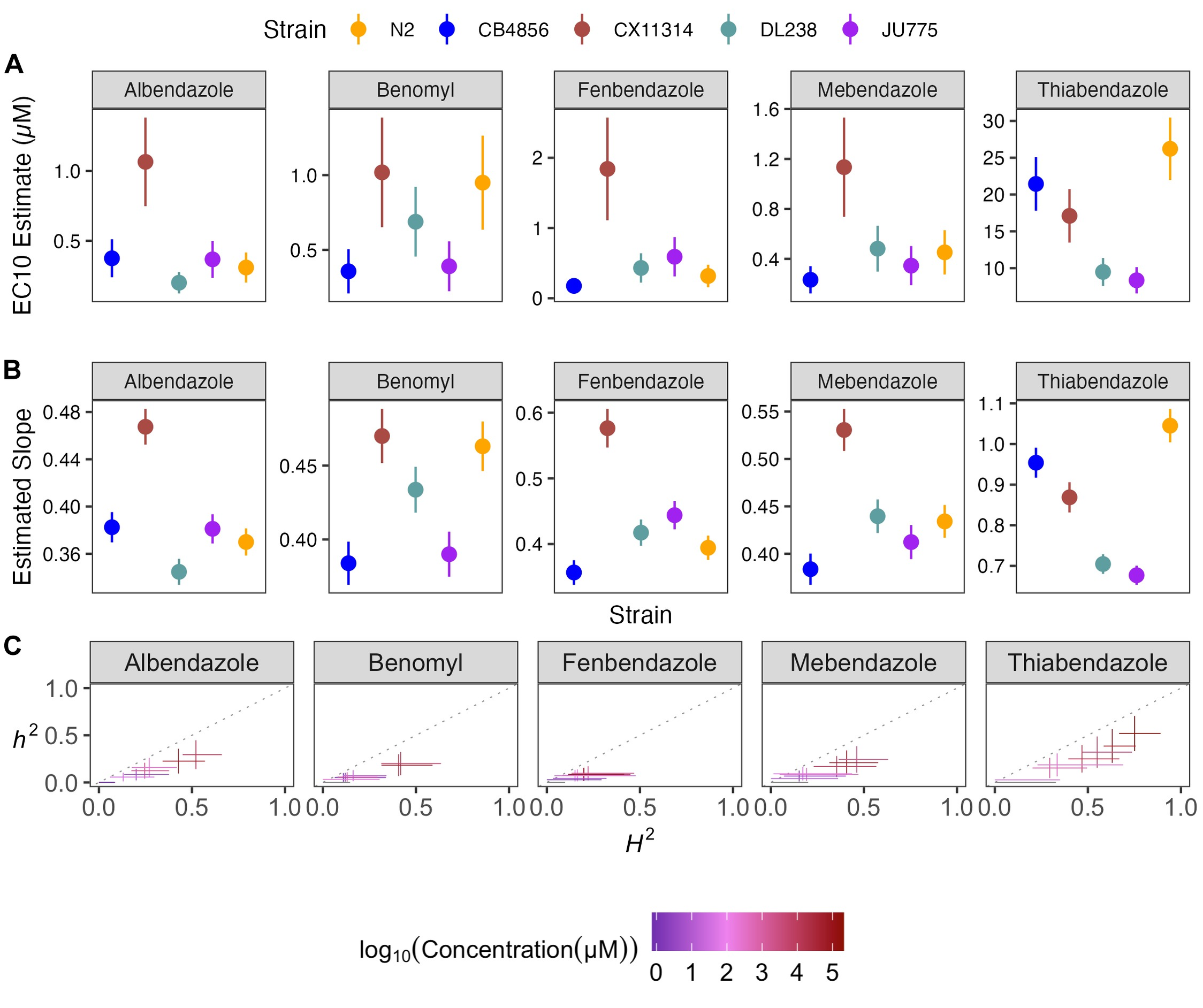

Supplement: S2 Fig — (A) Strain-specific EC10 estimates (e) for each benzimidazole are displayed for each strain. Standard errors for each strain- and anthelmintic-specific EC10 estimates are shown. (B) Strain-specific slope estimates (b) for each benzimidazole are displayed for each strain. Standard errors for each strain- and anthelmintic-specific slope estimate are indicated by the line extending vertically from each point. (C) The broad-sense (x-axis) and narrow-sense heritability (y-axis) of normalized animal length measurements were calculated for each concentration of each benzimidazole (Methods; Broad-sense and narrow-sense heritability calculations). The color of each cross corresponds to the log-transformed dose for which those calculations were performed. The horizontal line of the cross corresponds to the confidence interval of the broad-sense heritability estimate obtained by bootstrapping, and the vertical line of the cross corresponds to the standard error of the narrow-sense heritability estimate. (TIF) [file ppat.1011285.s008.tif]

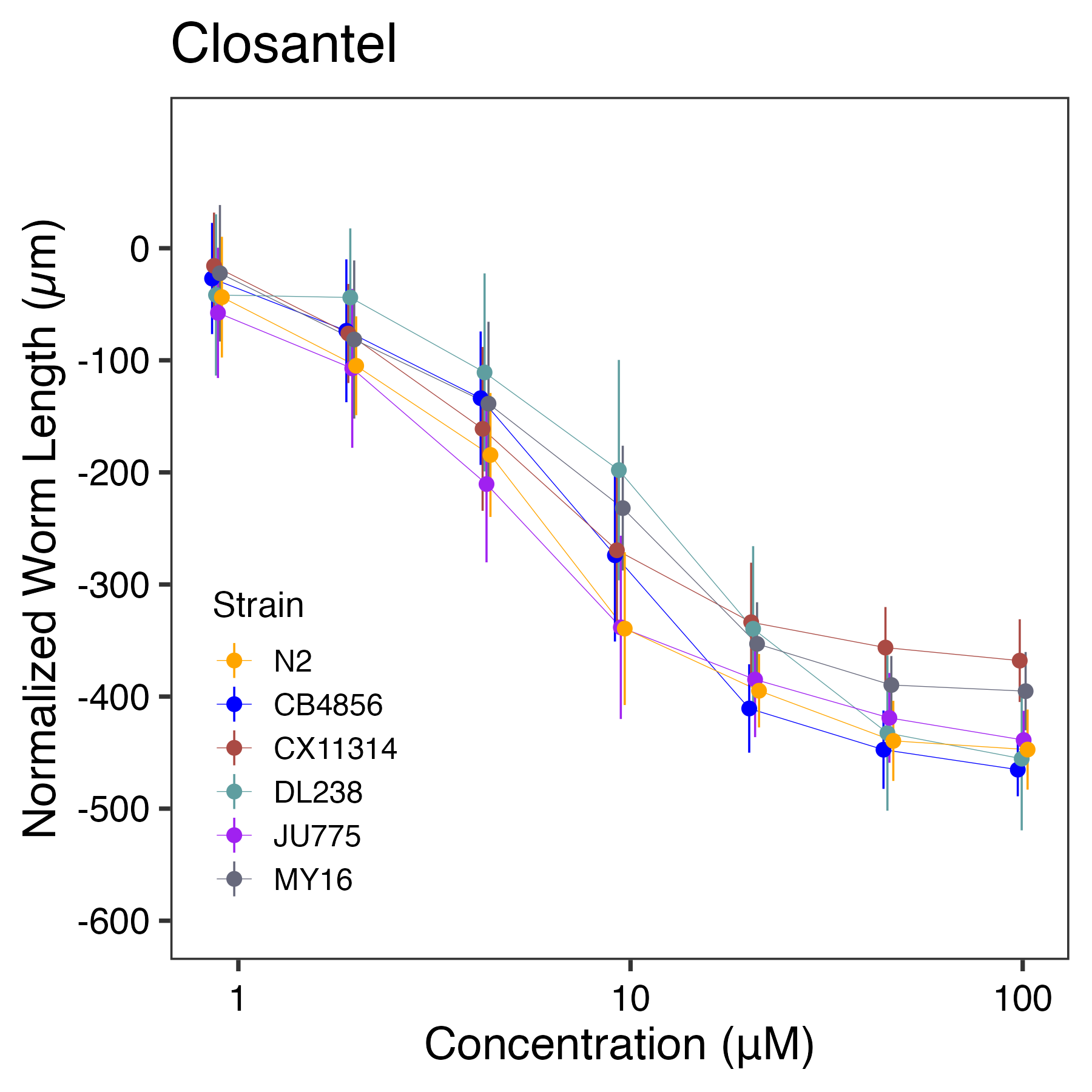

Supplement: S3 Fig — Normalized animal lengths (y-axis) are plotted for each strain as a function of the dose of closantel in the high-throughput microscopy assay (x-axis). Strains are denoted by color. Lines extending from points represent the standard deviations from the mean responses. Statistical normalization of animal lengths is described in Methods. (TIF) [file ppat.1011285.s009.tif]

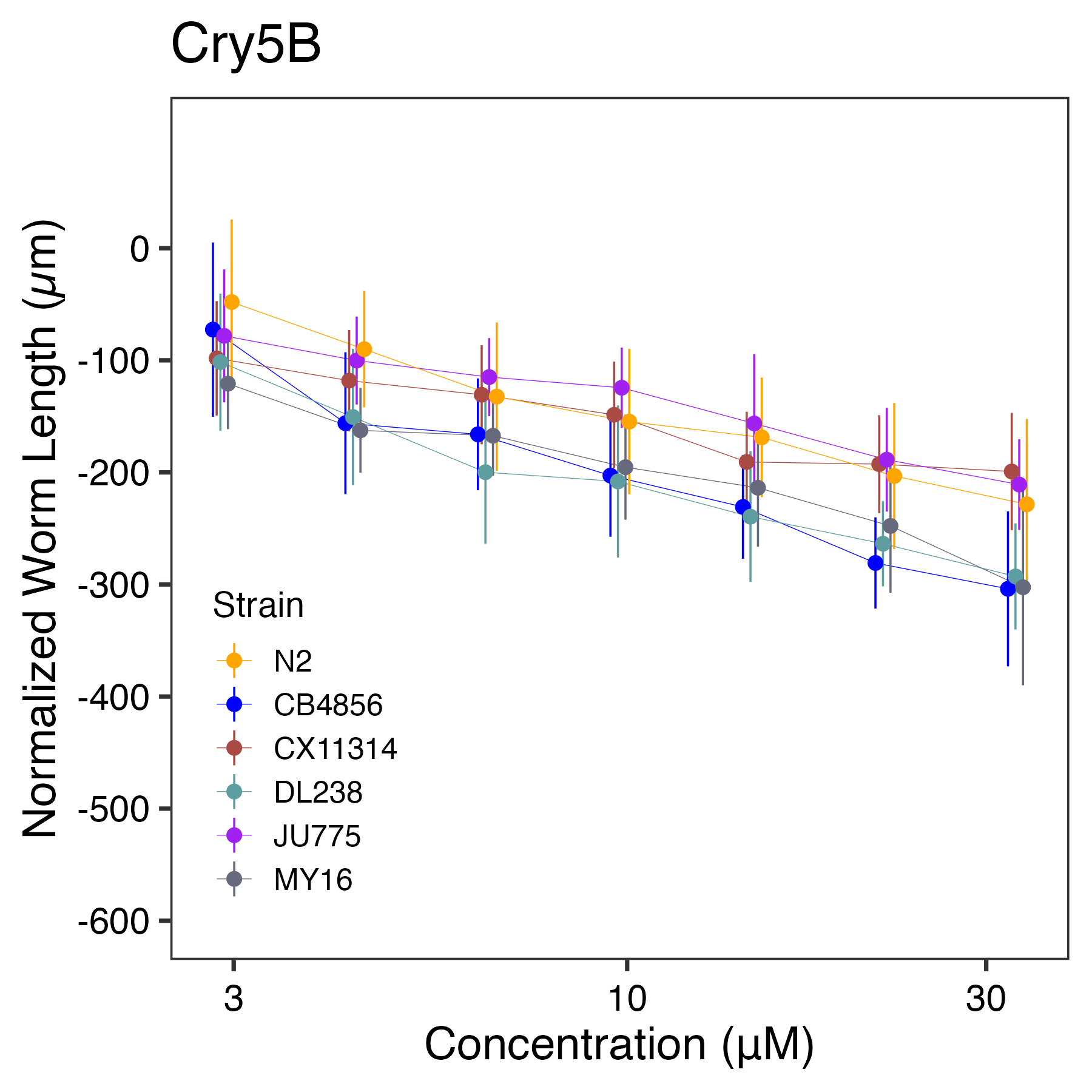

Supplement: S4 Fig — Normalized animal lengths (y-axis) are plotted for each strain as a function of the dose of Cry5B in the high-throughput microscopy assay (x-axis). Strains are denoted by color. Lines extending from points represent the standard deviations from the mean responses. Statistical normalization of animal lengths is described in Methods. (TIF) [file ppat.1011285.s010.tif]

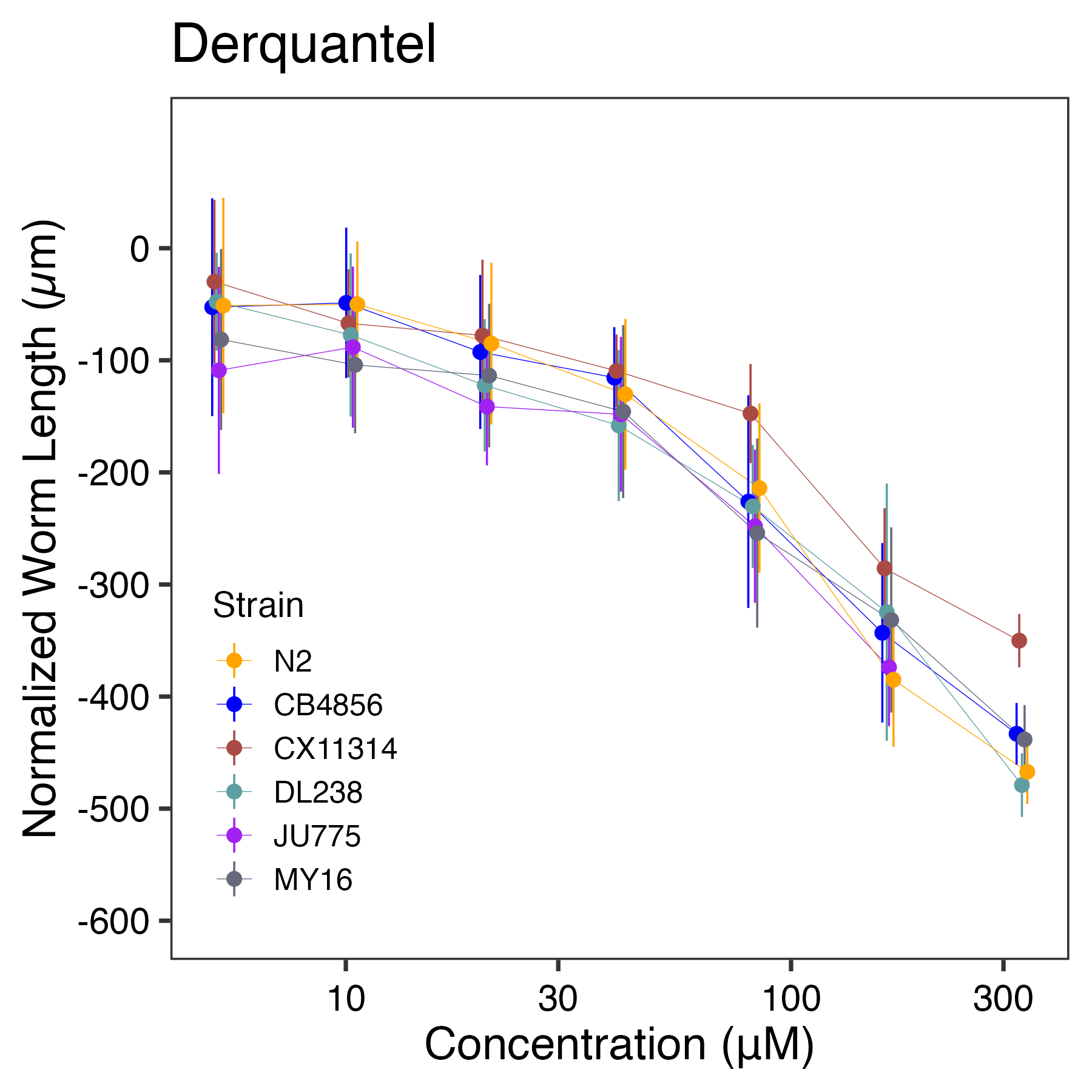

Supplement: S5 Fig — Normalized animal lengths (y-axis) are plotted for each strain as a function of the dose of derquantel in the high-throughput microscopy assay (x-axis). Strains are denoted by color. Lines extending from points represent the standard deviations from the mean responses. Statistical normalization of animal lengths is described in Methods. (TIF) [file ppat.1011285.s011.tif]

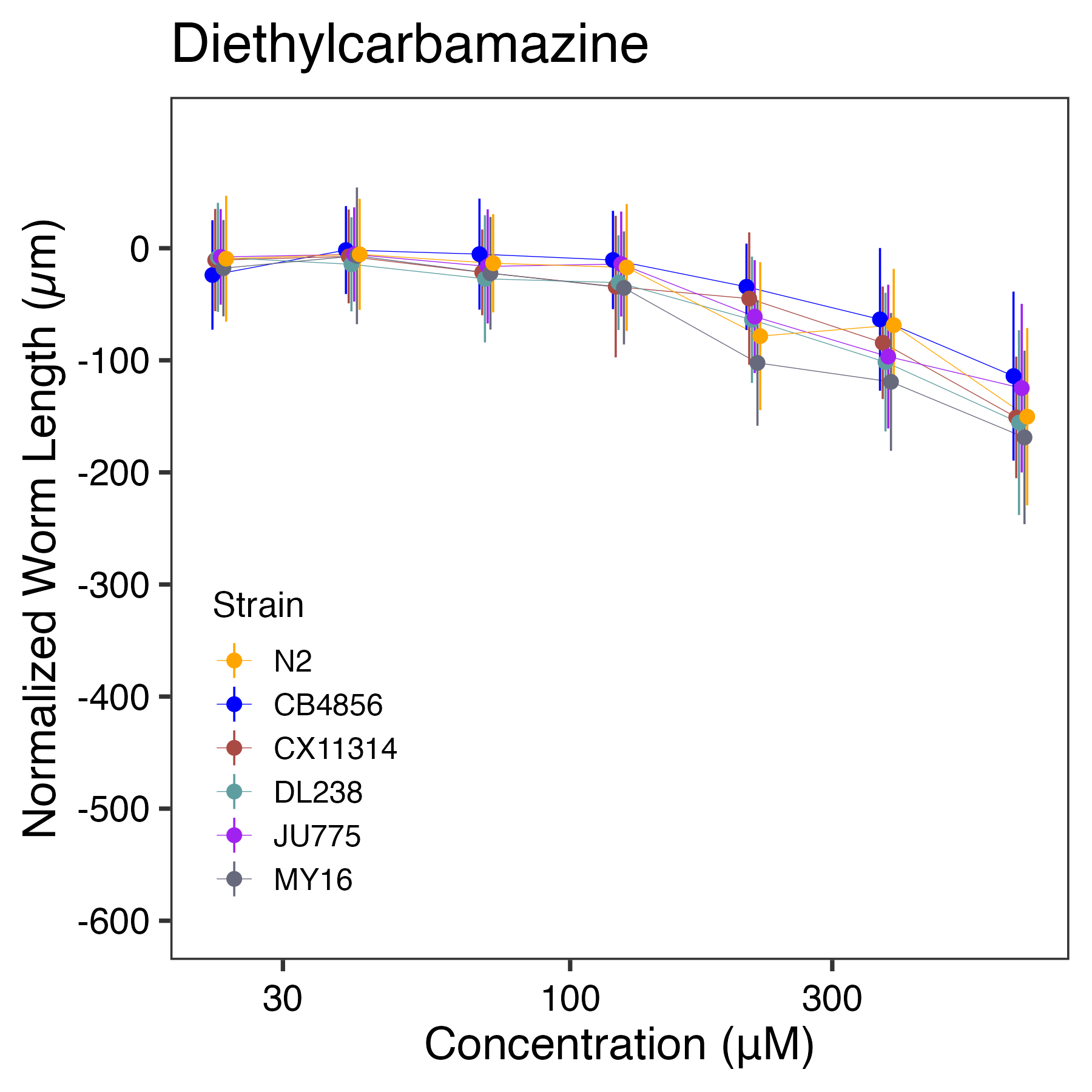

Supplement: S6 Fig — Normalized animal lengths (y-axis) are plotted for each strain as a function of the dose of diethylcarbamazine in the high-throughput microscopy assay (x-axis). Strains are denoted by color. Lines extending from points represent the standard deviations from the mean responses. Statistical normalization of animal lengths is described in Methods. (TIF) [file ppat.1011285.s012.tif]

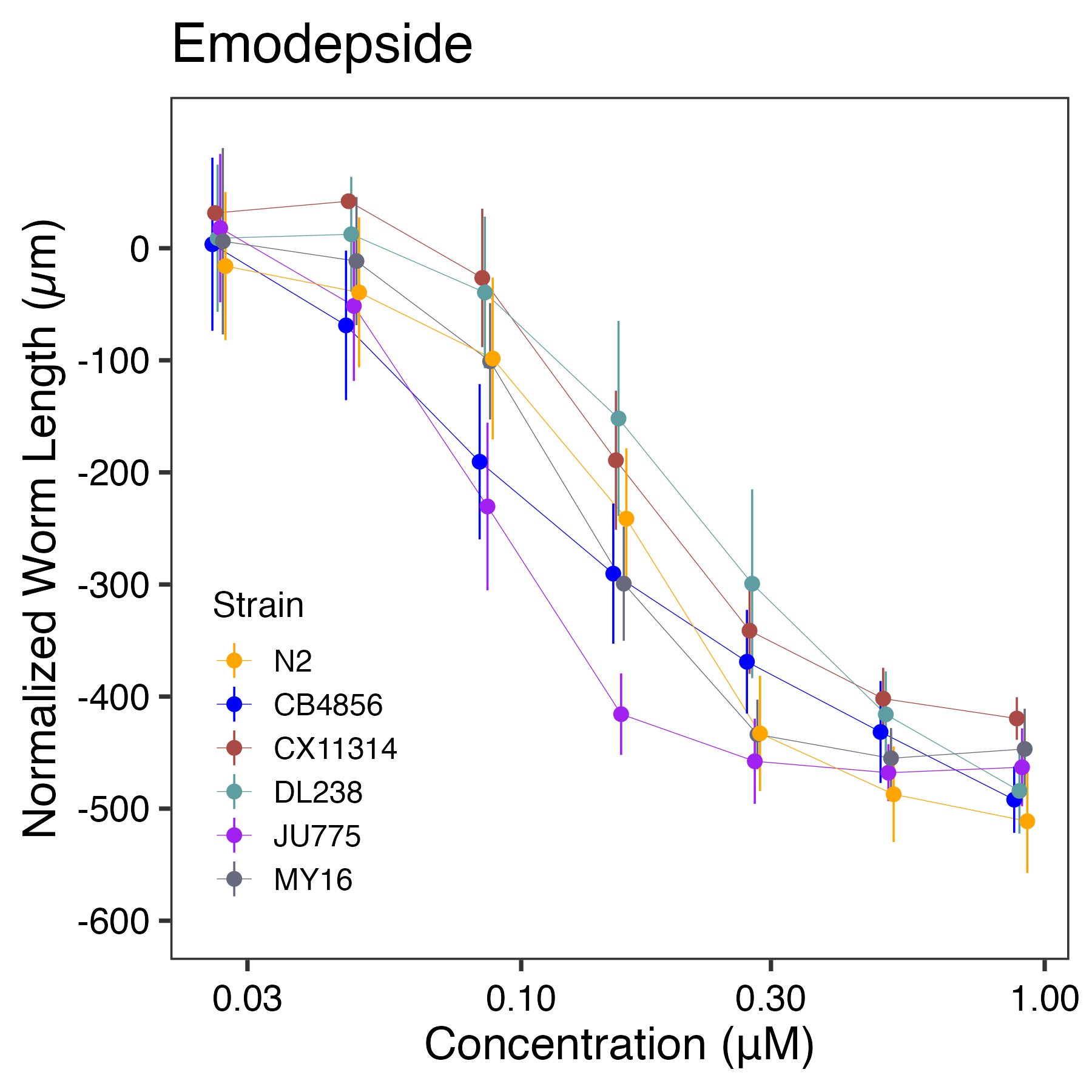

Supplement: S7 Fig — Normalized animal lengths (y-axis) are plotted for each strain as a function of the dose of emodepside in the high-throughput microscopy assay (x-axis). Strains are denoted by color. Lines extending from points represent the standard deviations from the mean responses. Statistical normalization of animal lengths is described in Methods. (TIF) [file ppat.1011285.s013.tif]

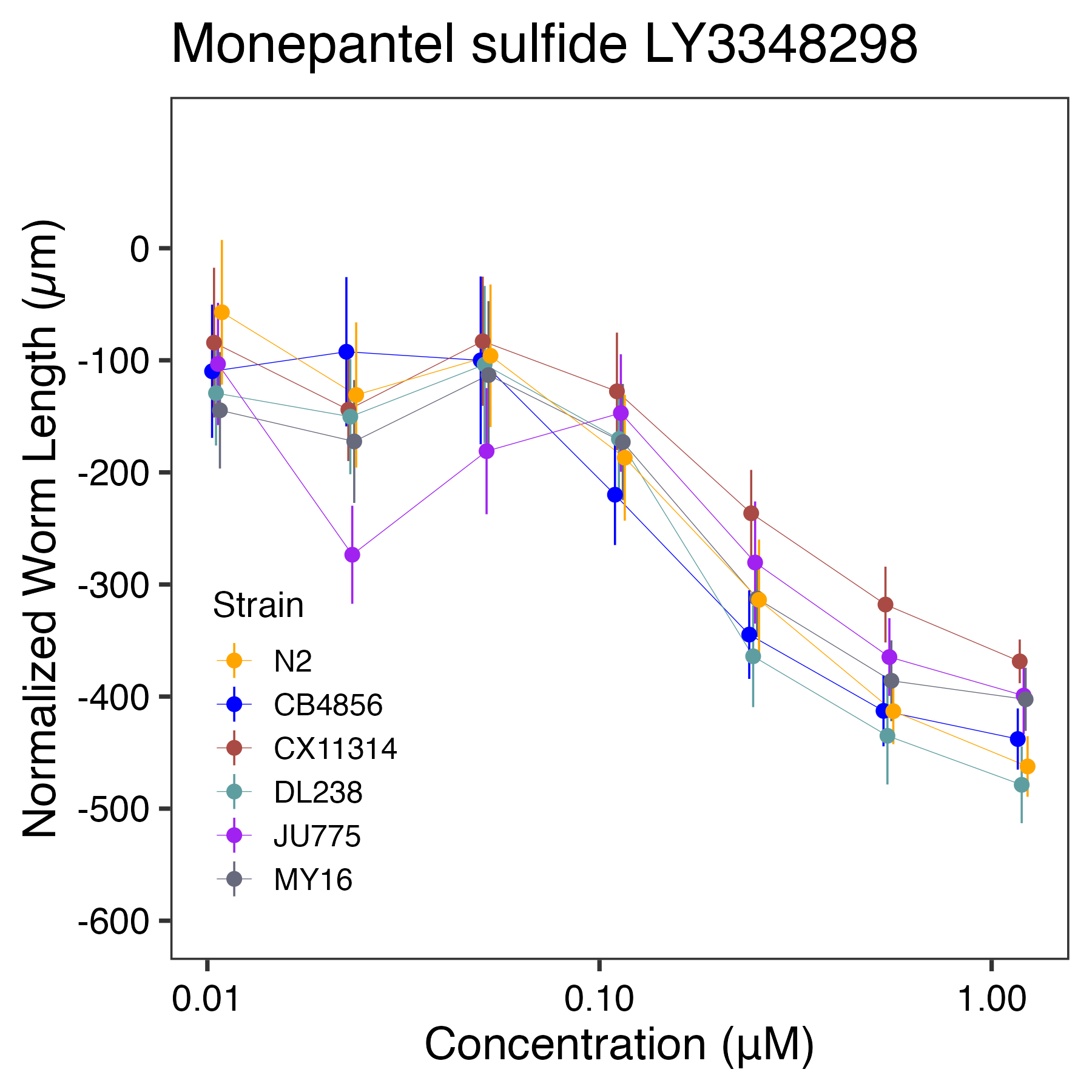

Supplement: S8 Fig — Normalized animal lengths (y-axis) are plotted for each strain as a function of the dose of monepantel sulfide in the high-throughput microscopy assay (x-axis). Strains are denoted by color. Lines extending from points represent the standard deviations from the mean responses. Statistical normalization of animal lengths is described in Methods. (TIF) [file ppat.1011285.s014.tif]

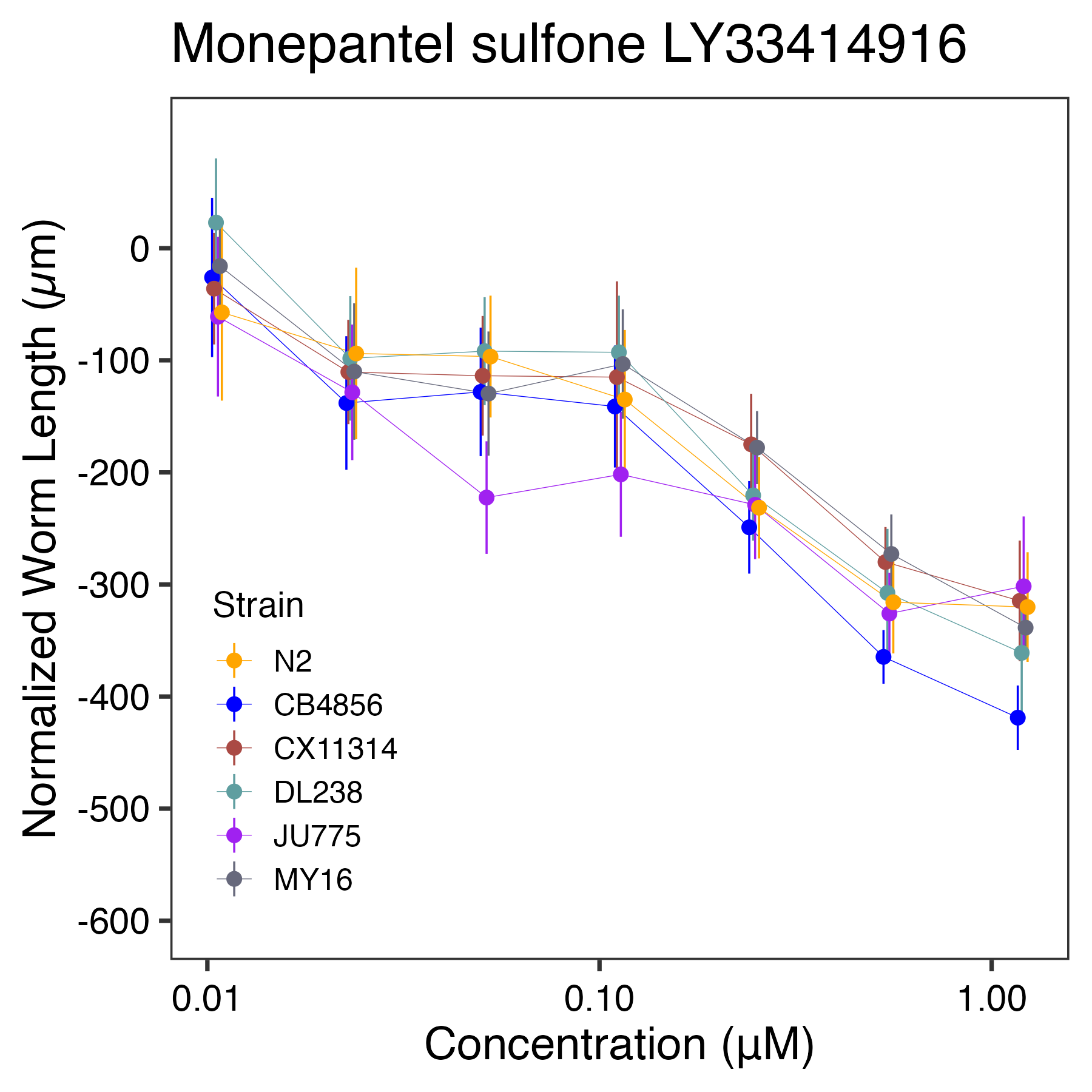

Supplement: S9 Fig — Normalized animal lengths (y-axis) are plotted for each strain as a function of the dose of monepantel sulfone in the high-throughput microscopy assay (x-axis). Strains are denoted by color. Lines extending from points represent the standard deviations from the mean responses. Statistical normalization of animal lengths is described in Methods. (TIF) [file ppat.1011285.s015.tif]

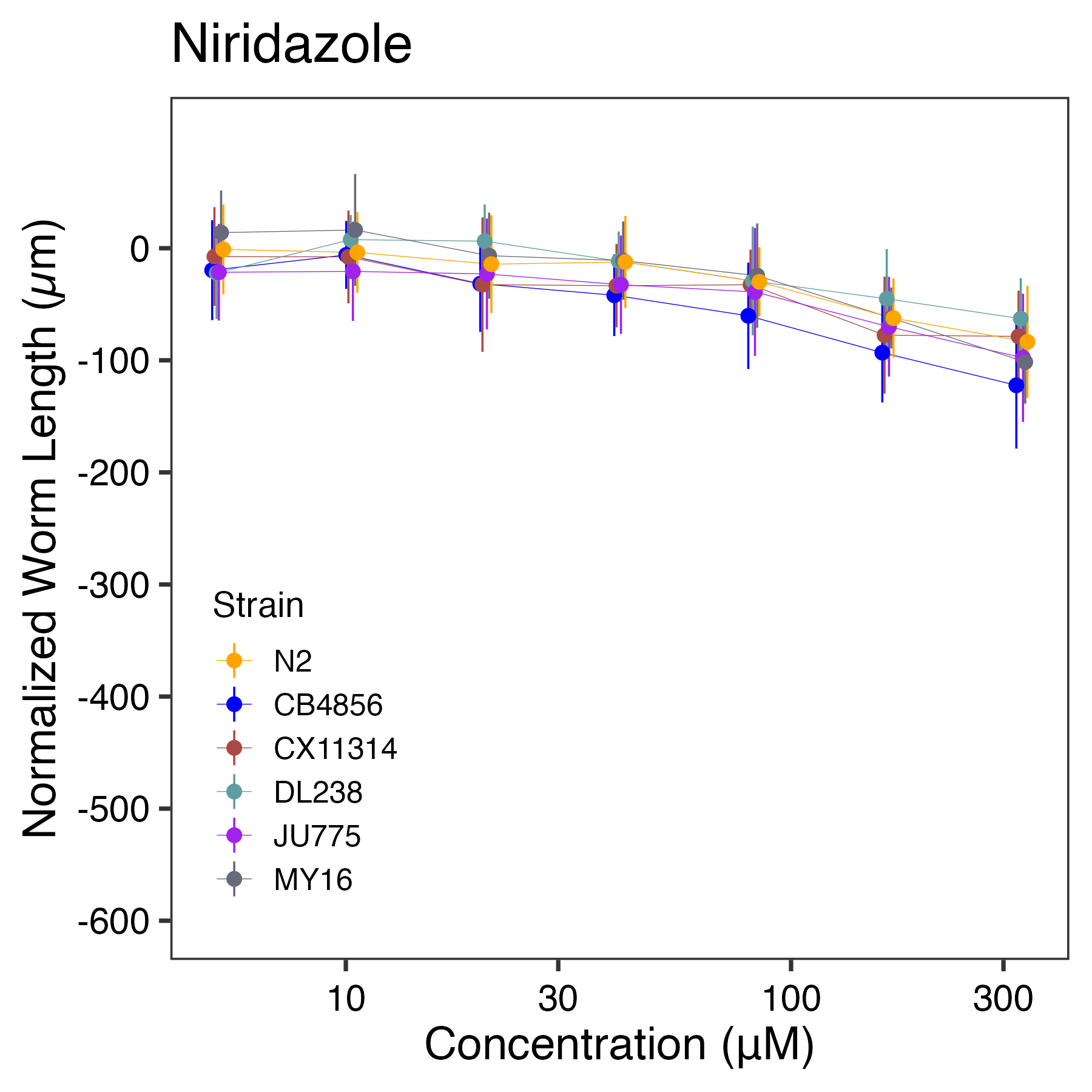

Supplement: S10 Fig — Normalized animal lengths (y-axis) are plotted for each strain as a function of the dose of niridazole in the high-throughput microscopy assay (x-axis). Strains are denoted by color. Lines extending from points represent the standard deviations from the mean responses. Statistical normalization of animal lengths is described in Methods. (TIF) [file ppat.1011285.s016.tif]

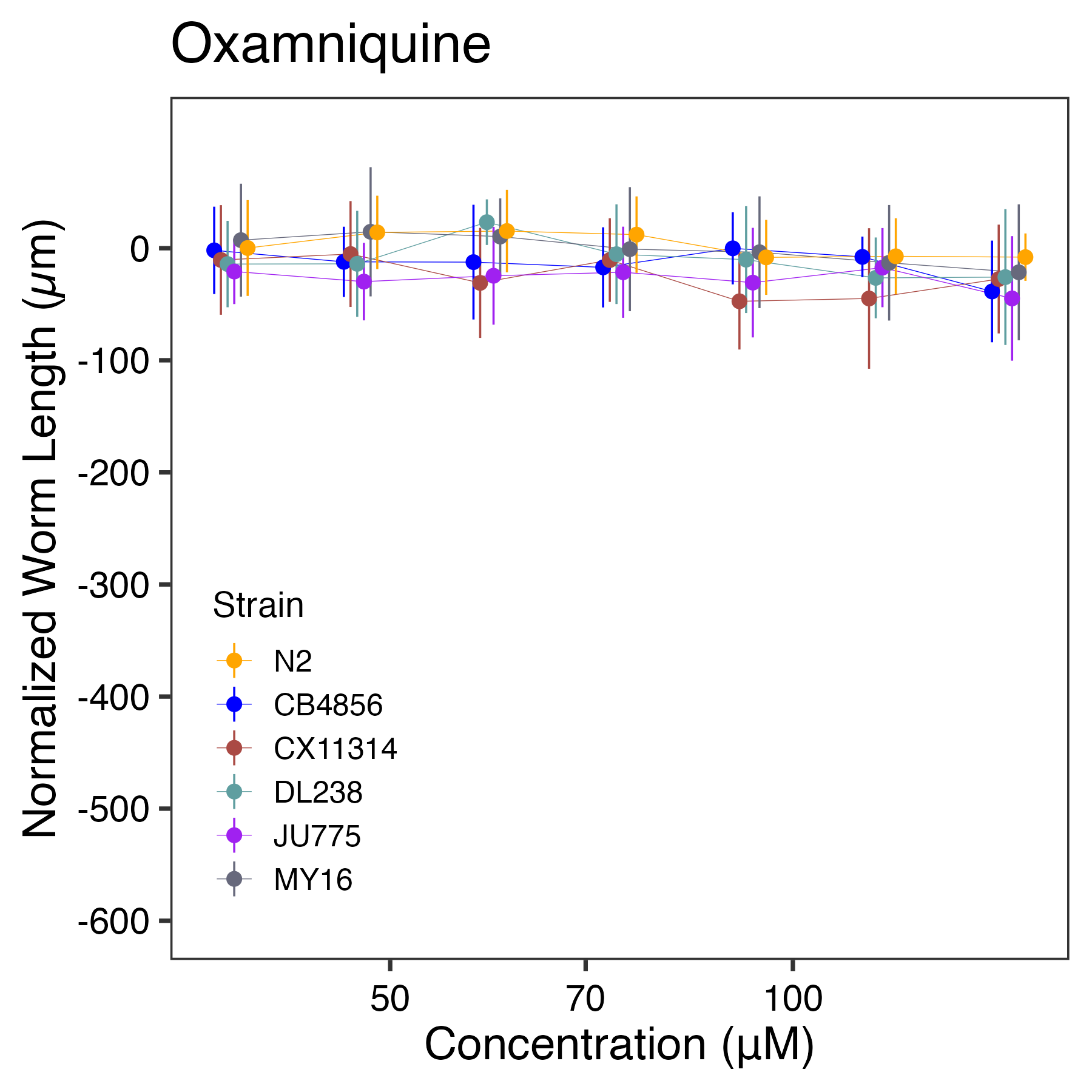

Supplement: S11 Fig — Normalized animal lengths (y-axis) are plotted for each strain as a function of the dose of oxamniquine in the high-throughput microscopy assay (x-axis). Strains are denoted by color. Lines extending from points represent the standard deviations from the mean responses. Statistical normalization of animal lengths is described in Methods. (TIF) [file ppat.1011285.s017.tif]

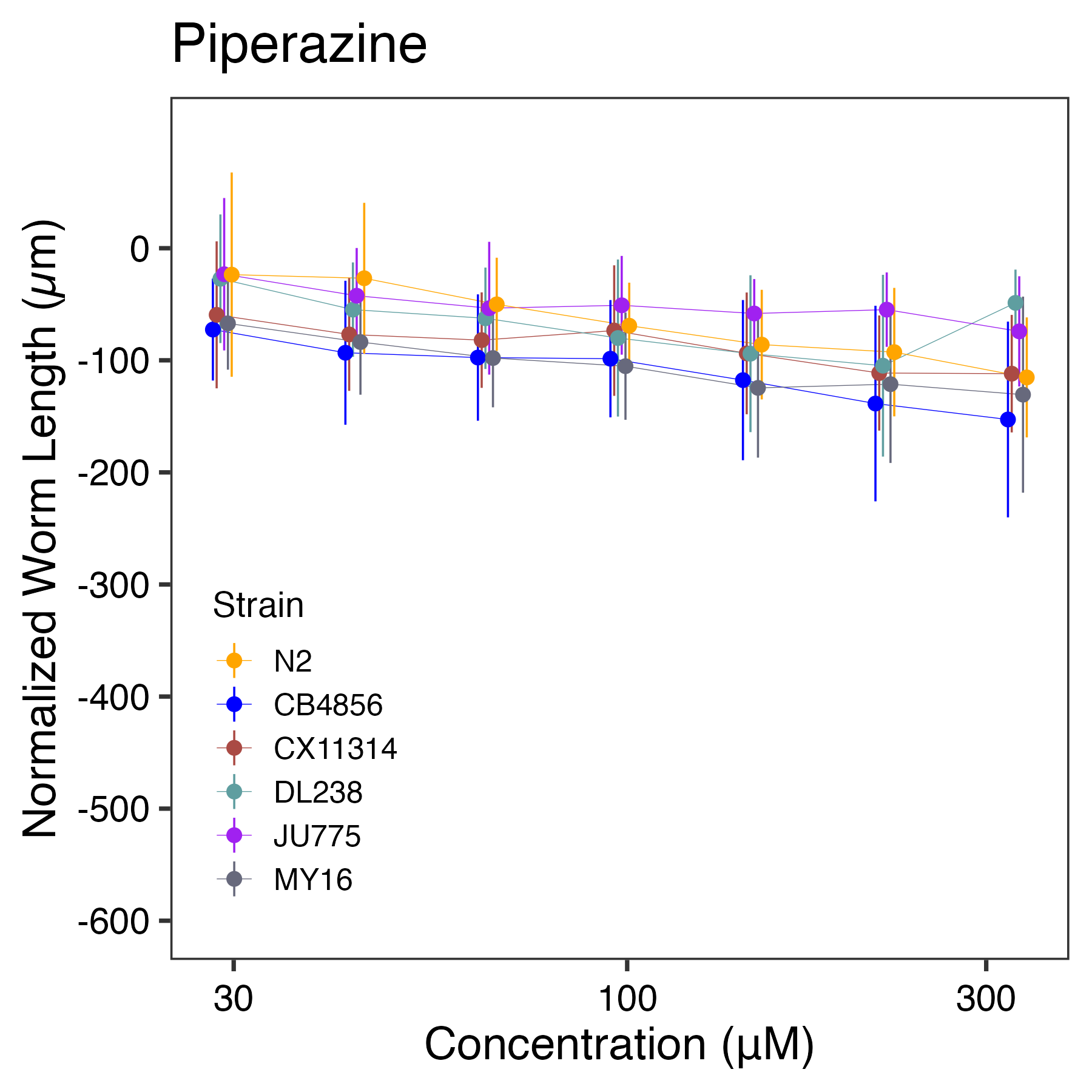

Supplement: S12 Fig — Normalized animal lengths (y-axis) are plotted for each strain as a function of the dose of piperazine in the high-throughput microscopy assay (x-axis). Strains are denoted by color. Lines extending from points represent the standard deviations from the mean responses. Statistical normalization of animal lengths is described in Methods. (TIF) [file ppat.1011285.s018.tif]

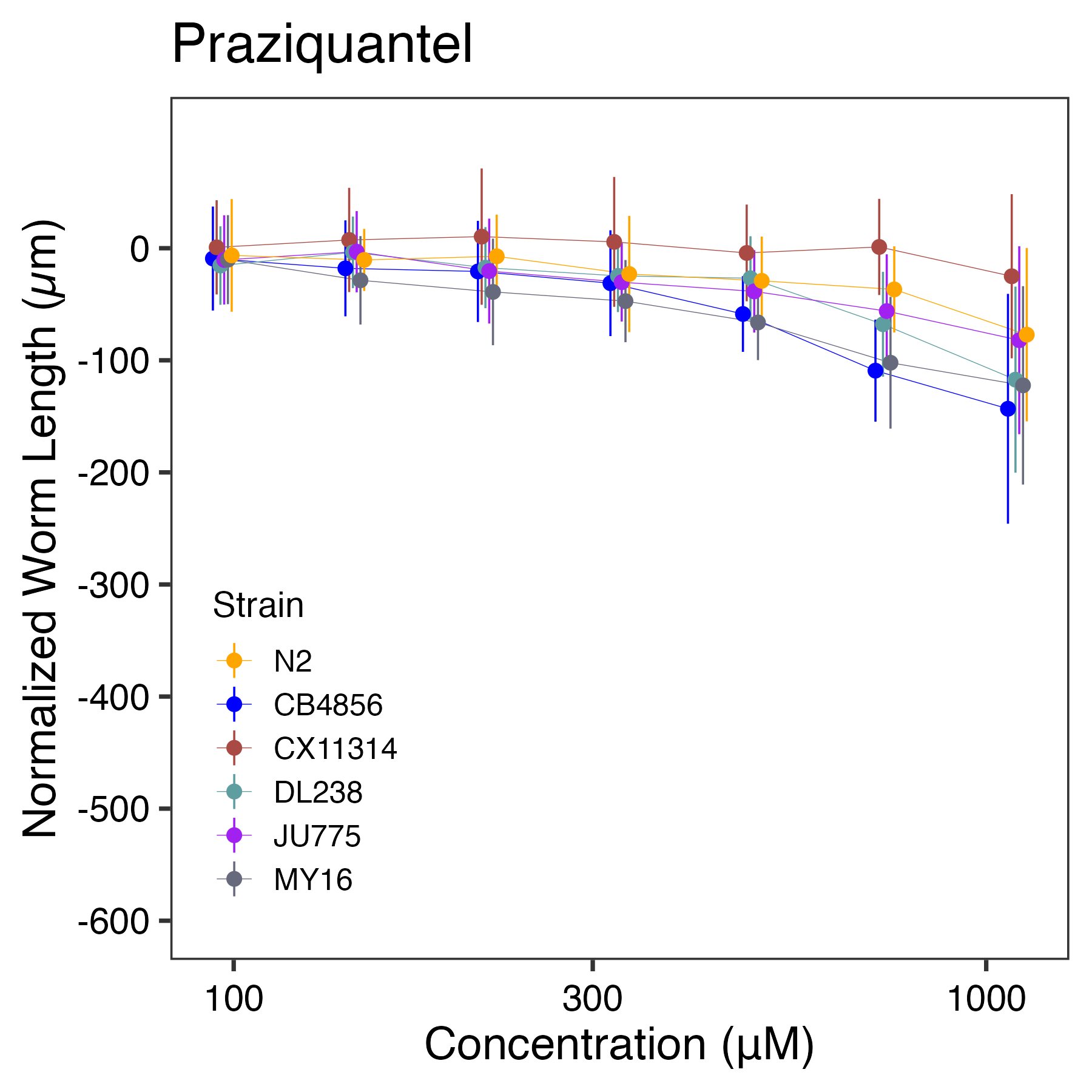

Supplement: S13 Fig — Normalized animal lengths (y-axis) are plotted for each strain as a function of the dose of praziquantel in the high-throughput microscopy assay (x-axis). Strains are denoted by color. Lines extending from points represent the standard deviations from the mean responses. Statistical normalization of animal lengths is described in Methods. (TIF) [file ppat.1011285.s019.tif]

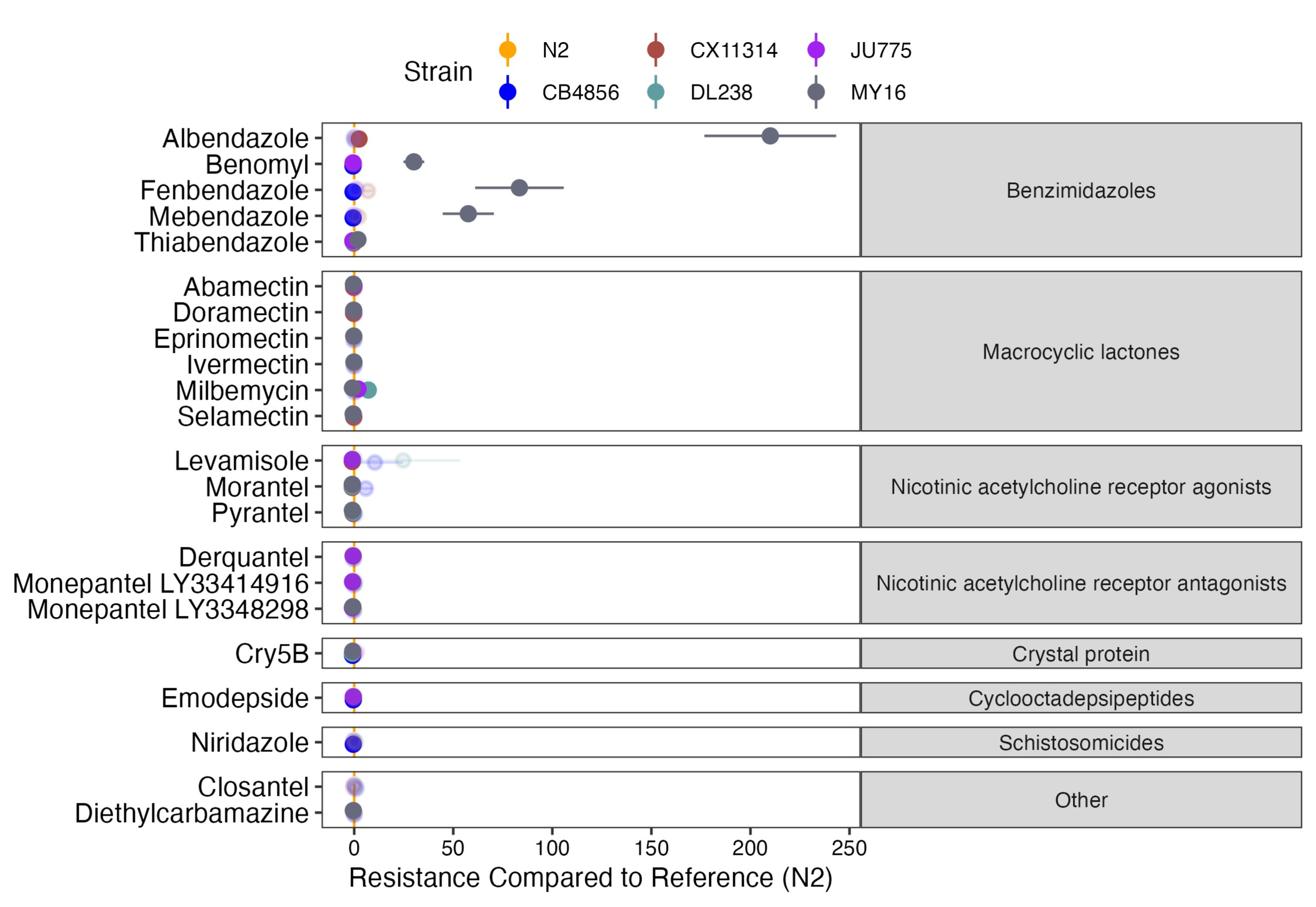

Supplement: S14 Fig — For each anthelmintic, the relative potency of that anthelmintic against each strain compared to the N2 strain is shown. Solid points denote strains with significantly different relative resistance to that anthelmintic (Student’s t-test and subsequent Bonferroni correction with a padj < 0.05), and faded points denote strains not significantly different than the N2 strain. The broad category to which each anthelmintic belongs is denoted by the strip label for each facet. Anthelmintic drugs with undefined EC10 estimates (estimates greater than the maximum dose to which animals were exposed) are not shown. (TIF) [file ppat.1011285.s020.tif]

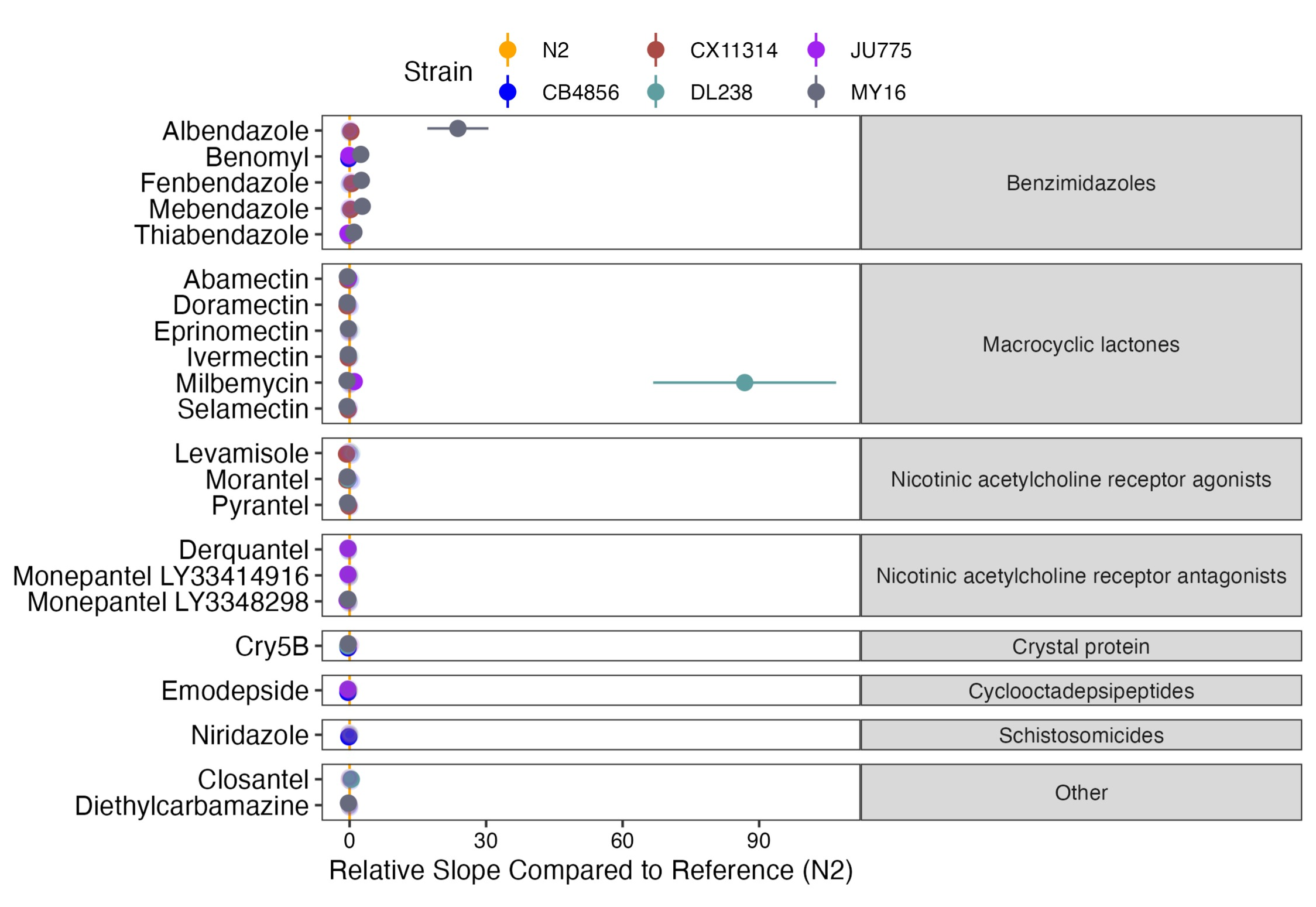

Supplement: S15 Fig — For each anthelmintic, the relative steepness of the dose-response slope inferred for that strain compared to the N2 strain is shown. Solid points denote strains with significantly different dose-response slopes (Student’s t-test and subsequent Bonferroni correction with a padj < 0.05), and faded points denote strains without significantly different slopes than the N2 strain. The broad category to which each anthelmintic belongs is denoted by the strip label for each facet. Anthelmintic drugs with undefined slope estimates are not shown. (TIF) [file ppat.1011285.s021.tif]

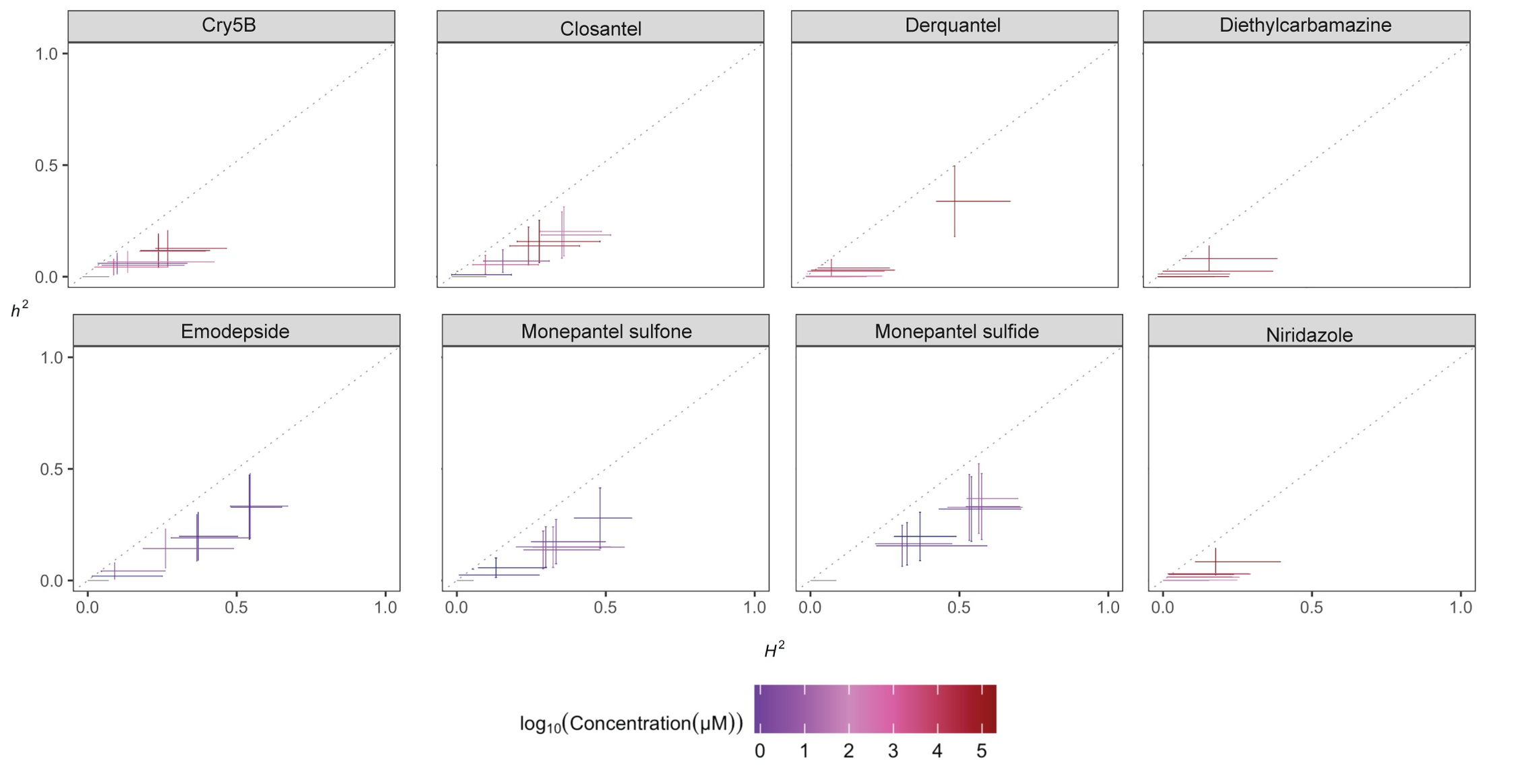

Supplement: S16 Fig — The broad-sense (x-axis) and narrow-sense heritability (y-axis) of normalized animal length measurements were calculated for each concentration of each nicotinic acetylcholine receptor agonist (Methods; Broad-sense and narrow-sense heritability calculations). The color of each cross corresponds to the log-transformed dose for which those calculations were performed. The horizontal line of the cross corresponds to the confidence interval of the broad-sense heritability estimate obtained by bootstrapping, and the vertical line of the cross corresponds to the standard error of the narrow-sense heritability estimate. Heritability could not be calculated for anthelmintics without an EC10. (TIF) [file ppat.1011285.s022.tif]

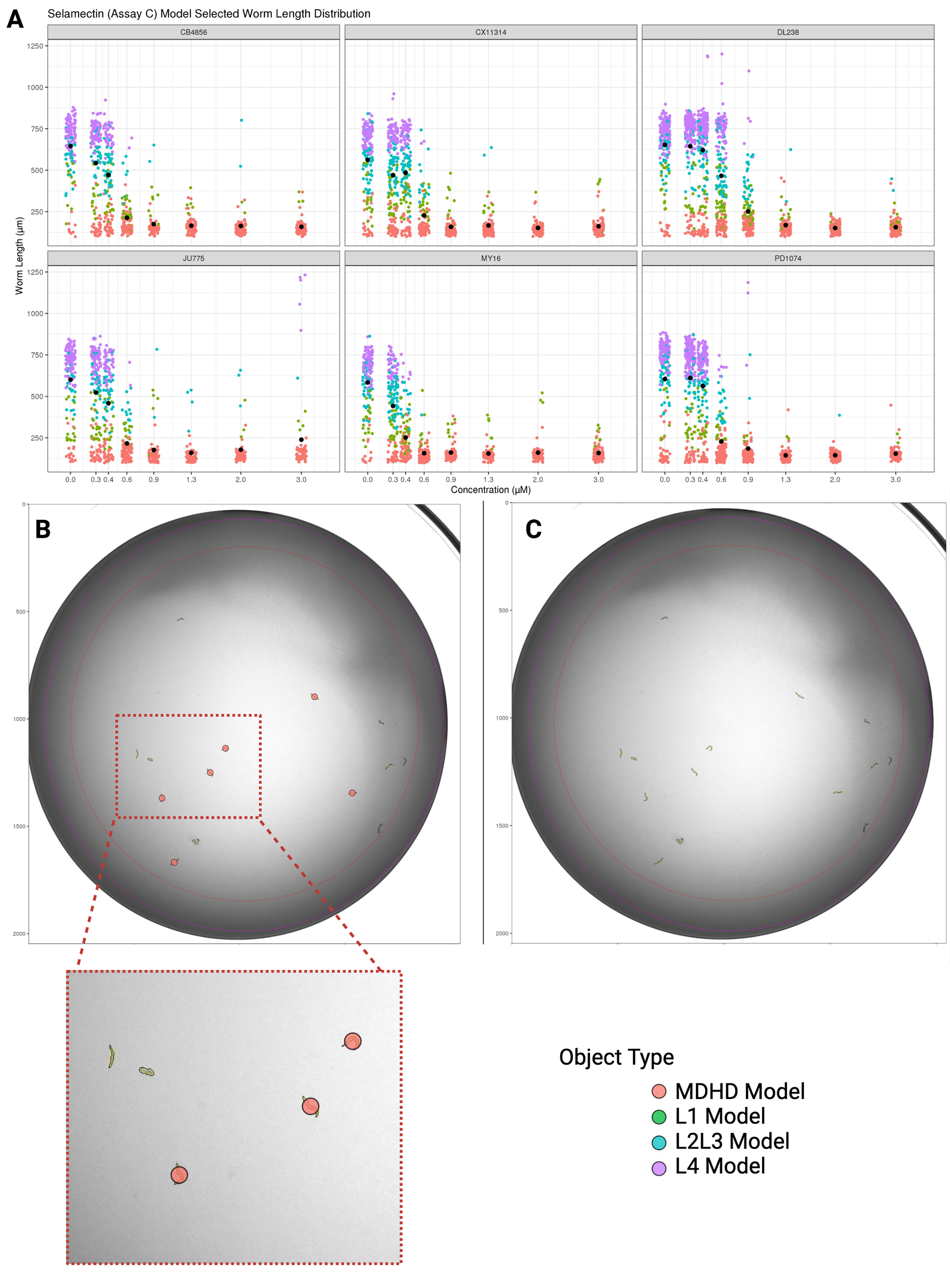

Supplement: S17 Fig — (A) Distribution of animals by length (y-axis) is plotted for each strain by dose (x-axis) for selamectin in Assay C. Each data point is a single observed object. Data points are colored by worm model: L4 larval stage (purple), L2 and L3 larval stages (turquoise), L1 larval stage (green), and MDHD (pink). Average worm length by dose is denoted by the black datapoint. Selamectin high dose wells are shown for both the (B) Worm_Length > 30-pixel filter and (C) Worm_Length > 50-pixel filter. The inset shows small animals recognized by the MDHD model. Pink overlays indicate an MDHD model selected object. Animals with no overlay are filtered out during the cleaning steps. see Methods for details on data cleaning metrics. (TIF) [file ppat.1011285.s023.tif]

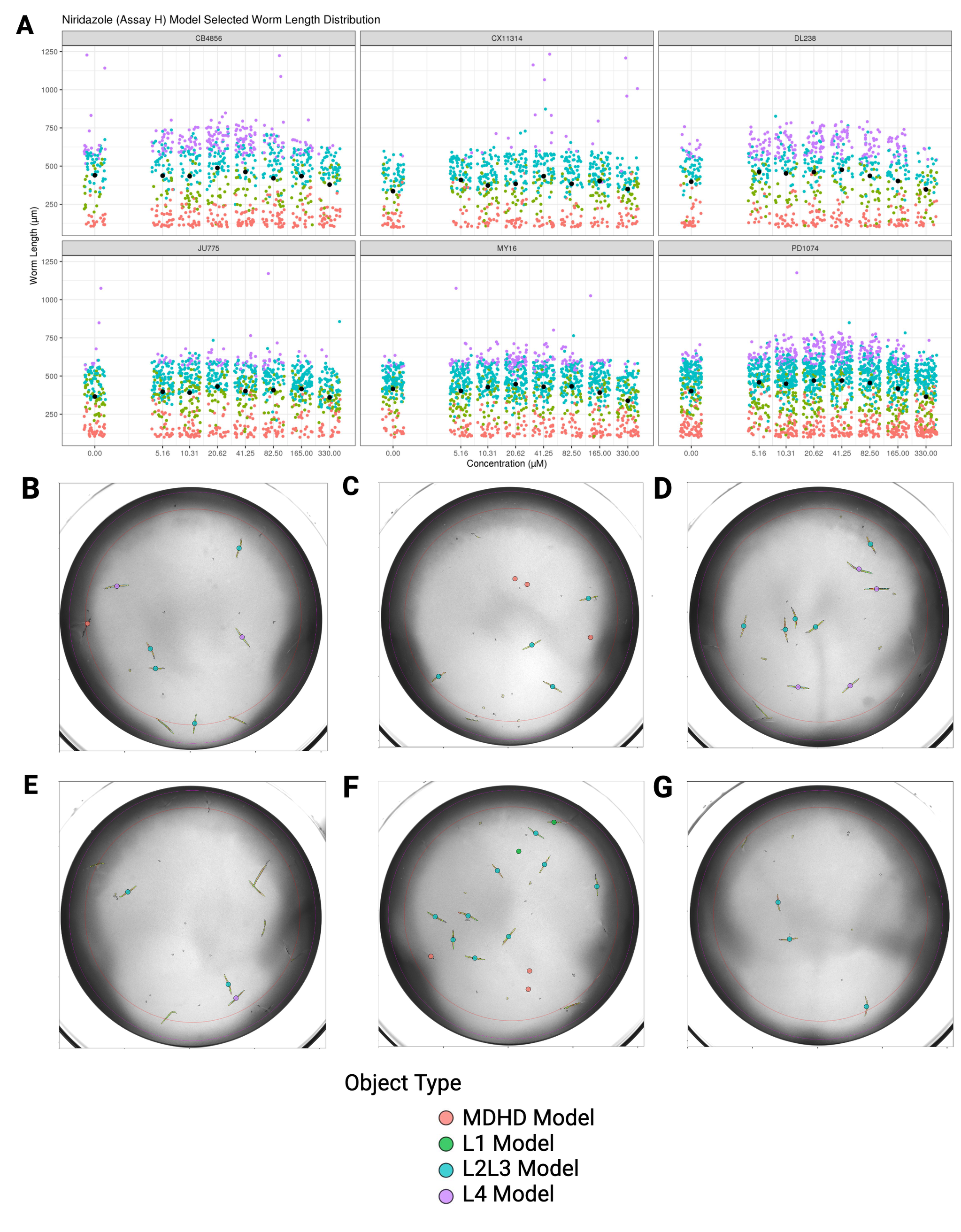

Supplement: S18 Fig — (A) Distribution of animals by length (y-axis) is plotted for each strain by dose (x-axis) for niridazole in Assay H. Each data point is a single observed object. Data points are colored by worm model: L4 larval stage (purple), L2 and L3 larval stages (turquoise), L1 larval stage (green), and MDHD (pink). The black data point denotes the average worm length by dose. Well images obtained in Assay H for niridazole dose-response experiments are representative of problematic control well features caused by the presence of small animals categorized by the L1 model and L2L3 for each strain: (B) CB4856, (C) CX11314, (D) DL238, (E) JU775, (F) MY16, and (G) N2. (TIF) [file ppat.1011285.s024.tif]
