# Supplementary material for: Variation in anthelmintic responses are driven by genetic differences among diverse C. elegans wild strains
Source: PLoS Pathog. 2023 Apr 3;19(4):e1011285. doi: 10.1371/journal.ppat.1011285 (PMC10101645; doi:10.1371/journal.ppat.1011285)
Supplement: Attachment — Submitted filename: Response_to_PLOS_Pathogens_review.docx [file ppat.1011285.s025.docx]

Dear Editor,

Thank you for handling our manuscript “Variation in anthelmintic responses are driven by genetic differences among diverse *C. elegans* wild strains”. We have addressed all of the editor’s and the reviewers’ critiques. The manuscript is vastly improved. Please find their comments in bold below, with our responses in normal face font. Changes to the manuscript are included as quotes below our responses.

**Editor’s comments to the author:**

**#1. PloS Pathogens is not a genetics journal; a clearer explanation of the relevance of measuring heritability (H2 and h2) is needed. I suggest a clear statement of this in the introduction. In particular, the manuscript uses genomic sequence data from the C.elegans lines used to estimate SNP-based heritability. This is distinct from the more commonly understood heritability estimation methods using pedigrees or twins and also needs some explanation for non-genetics audience. I was personally surprised that accurate measures of SNP heritability can be derived from just 6 C.elegans lines - please can you provide further demonstrations that this is possible and expected from the literature?**

Thank you for this critique. We believe that our updated explanation and edits to the manuscript improve the heritability description and relevance.

The genotype matrix used in the heritability calculations was generated using the Genomatrix Profile in NemaScan (https://github.com/AndersenLab/NemaScan), a genome-wide association mapping and simulation software for *C. elegans* and other *Caenorhabditis* species. NemaScan uses the GCTA software suite to calculate genomic relationship matrices [[1,2]](https://paperpile.com/c/8HQFcn/6Dug+eqk2). A hard-filtered VCF file ([WI.20220216.hard-filter.vcf.gz](https://storage.googleapis.com/caendr-site-public-bucket/dataset_release/c_elegans/20220216/variation/WI.20220216.hard-filter.vcf.gz)) from the latest release of CeNDR (<https://www.elegansvariation.org/data/release/latest>) was used when performing the Genomatrix Profile. The hard-filtered VCF file includes high-quality variants. A total of 550 isotypes, genetically distinct strains, were used when calculating the genotype matrix. Although we focused on the heritability of the six strains used in our dose-response assays, 550 isotypes were used to generate the genotype matrix.

We have modified the manuscript in the following ways to make our heritability methods clear:

- *Abstract:* In the *Abstract*, we have briefly defined heritability which reads:

“Third, we quantified the levels of anthelmintic response variation caused by genetic differences among individuals (heritability) to each drug and observed a significant correlation between exposure closest to the EC_10_ and the exposure that exhibited the most heritable responses.”

- *Introduction:* We defined heritability in the *Introduction* as suggested. This section now reads:

“Over-reliance and inappropriate use of these anthelmintics have placed strong selective pressures on parasites and caused the evolution of anthelmintic resistance (AR) in every drug class [3]. In many cases, AR is highly heritable, which suggests that genetics plays an important role in the evolution of resistant nematodes [5,6]. Therefore, calculating the heritability of AR allows us to estimate the fraction of phenotypic variation in anthelmintic responses that can be explained by genetic variation in a population [7]. The narrow-sense heritability is a measure of the genetic component that is contributed by alleles that act additively so it can be used to calculate how much AR across a population will increase when mating resistant parents [6]. Because AR is highly heritable, we can identify the genes involved in AR and model resistance over time. These measures are critical because AR causes the drug to become ineffective [4]. With this knowledge, we can responsibly apply drugs and implement treatment strategies to reduce our global infection rate and burden of parasitic nematodes.”

- Methods: In the *Methods* subheading *Broad-sense and narrow-sense heritability calculations,* we further defined the different types of heritability and described how we generated the genomatrix profile. This section now reads:

**“Broad-sense and narrow-sense heritability calculations**

Phenotypic variance can be split into two parts, genetic variance (*V_G_*) and residual variance (*V_E_*). To determine the genetic contributions to phenotypic variance, we measured broad-sense (*H^2^*) and narrow-sense heritability (*h^2^*). Broad-sense heritability (*H^2^*) is the total fraction of phenotypic variance explained by genetic variation in a population [7]. Here, *H^2^* was calculated using the equation, *H^2^ = V_G_ / (V_G_+V_E_)*, where we extracted the among strain variance (*V_G_*) and residual variance (*V_E_*). Broad-sense heritability was estimated (*H^2^*) using in the R statistical environment (version 4.0.4) using the R package *lme4* (v1.1-27.1) to fit a linear mixed-effects model to the normalized median animal length data using strain as a random effect [25]. Genetic variance (*V_G_*) can be partitioned into additive (*V_A_*) and non-additive (*V_NA_*) variance components. Additive genetic variance (*V_A_*) is the amount of genetic variance explained by genotyped variants in a specific population. Narrow-sense heritability (*h^2^*) is the fraction of phenotypic variation explained by additive genetic variation in a population [7]. Narrow-sense heritability (*h^2^*) measures the additive genetic variance (*V_A_*) over the total phenotypic variance, *h^2^ = V_A_ / V_P_*. To calculate *h^2^*, we first generated a strain matrix using the strain *genomatrix profile* on NemaScan (https://github.com/AndersenLab/NemaScan), a genome-wide association (GWA) mapping and simulation pipeline [73]. NemaScan uses the GCTA software suite to calculate genomic relationship matrices [74,75] and used the hard-filtered variant call format (VCF) file ([WI.20220216.hard-filter.vcf.gz](https://storage.googleapis.com/caendr-site-public-bucket/dataset_release/c_elegans/20220216/variation/WI.20220216.hard-filter.vcf.gz)) generated in the 20220216 CeNDR release (<https://www.elegansvariation.org/data/release/latest>). The VCF used includes high-quality variants from 550 *C. elegans* isotypes, genetically distinct strains, after quality control filters. We then calculated *h^2^* using the *sommer* (v4.1.3) R package by calculating the variance-covariance matrix (*M_A_*) from the strain matrix using the *sommer::A.mat* function [25]. We estimated *V_A_* using the linear mixed-effects model function *sommer::mmer* using strain as a random effect and *M_A_* as the covariance matrix. We then estimated *h^2^* and its standard error using the *sommer::vpredict* function (**S16 Fig**). Lastly, to identify the amount of additive genetic variance contributing to *h2*, a correlation between the *h2* of the exposure closest to the estimated EC_10_ and the drug doses that exhibited the maximum *h2* for each anthelmintic with definitive EC_10_ estimates was performed (**S1 Fig**).”

We hope that these edits make our heritability methods clear.

**#2. Two reviewers felt that the 48 hr assays examining impact of drugs on worm growth may not be comparable to measures of variation in drug resistance in parasitic helminths.**

Thank you for the comment. We agree that *C. elegans* and parasitic helminths differ. Additionally, most parasitic helminth species differ from each other using *in vivo* vs. *in vitro* experiments that also differ. Importantly, we do not know the variation of drug resistance in parasitic helminths. Our best understanding of how parasitic helminths respond to anthelmintics is from *Haemonchus contortus,* and we have many knowledge gaps even within this system.

However, we can use *C. elegans* as a free-living nematode model to assess nematode response to anthelmintics and identify genes and mechanisms involved in anthelmintic resistance. The following review, which is cited, highlights many *C. elegans* assays using the same 48-hour assay to measure the phenotypic response (body length) to various anthelmintics and toxicants: Hahnel, Steffen R., Clayton M. Dilks, Iring Heisler, Erik C. Andersen, and Daniel Kulke. "*Caenorhabditis elegans* in anthelmintic research–Old model, new perspectives." *International Journal for Parasitology: Drugs and Drug Resistance* 14 (2020): 237-248 and relates those traits to parasites. We did not want to repeat those arguments here.

In this manuscript, a 48-hour assay was used to measure a reduction in growth from the L1 to the L4 larval stage in *C. elegans*. Similar assays are not possible in many parasitic helminths, as many require a host for survival, but the trait is similar to a larval development assay (LDA). Here, we intend to highlight the information that can be taken from *C. elegans* to create more robust methods when performing assays or treatments for parasitic helminths. The assay in this manuscript allows us to (A) identify which anthelmintics are adequate nematicides in *C. elegans*, (B) show the variation in response across doses within and across anthelmintics, and (C) show that the diversity within the *C. elegans* species contributes to the variation in anthelmintic response and thus parasitic helminths would be expected to have even more variable responses.

We highlight these points in the following places in the manuscript:

- *Introduction*, second paragraph:

“Most of our knowledge about mechanisms of resistance comes from studies of a single strain within a single species, the laboratory-adapted strain of *Caenorhabditis elegans* called N2. However, a single genetic background, whether in a free-living or parasitic species, cannot capture the enormous diversity present in the entire species, nor can it predict how natural populations of parasitic nematodes will respond to a drug.”

“It is difficult to accurately test AR in genetically diverse parasitic nematodes because of multiple factors: a lack of access to relevant life cycle stages, lack of global sample collections, host-dependent and cost-intensive laboratory life cycles, complex or non-existent *in vitro* culture systems, and a limited molecular toolkit.”

- In the *Methods*, we made it clearer that we are measuring development from the L1 larval stage to the L4 larval stage. We now state:

“Next, 25 µl of the food and anthelmintic mixture was transferred into wells of 96-well microplates to feed the arrested L1s at a final HB101 concentration of OD_600_10 and expose L1 larvae to an anthelmintic at one of eight levels of the dilution series.”...

“Nematodes grown under control conditions or those unperturbed by an anthelmintic developed at a normal rate to the L4 larval stage. Nematodes affected by anthelmintics have delayed development.”

- In the *Results and Discussion*, we added:

“Differences in responses were measured by the change in developmental rate, as measured by animal length, a trait similar to larval development assay (LDA) [22]”

We hope these additions highlight our rationale for the 48-hour development experiment and clarify our methods.

**#3. There was also a concern that "contractile" responses of worms to drug exposure might confound this development-based assay - please provide further justification for the approaches used.**

Thank you for the opportunity to clarify. We have addressed the concern about contractile responses to Reviewer #2 (see Comment #9 under Part I - Summary).

Briefly, although BZs and NAChR agonists, for example, do elicit muscle contractions, we can still adequately measure body length. After *C. elegans* have been exposed to anthelmintics for 48 hours, each well is treated with 50 mM sodium azide for 10 minutes to paralyze and strengthen nematodes prior to imaging. Therefore, any worms that might have been contracted will be straightened prior to imaging. We describe the details of this assay in the *Methods* under the subheading “*High-throughput anthelmintic dose-response assay*.” This section reads as follows:

“After 48 hours of incubation in the presence of food and anthelmintic, the 96-well microplates were removed from the incubator and treated with 50 mM sodium azide in M9 for 10 minutes to paralyze and straighten nematodes. Images of nematodes in the microplates were immediately captured using a Molecular Devices ImageXpress Nano microscope (Molecular Devices, San Jose, CA) using a 2X objective.”

**#4. Similarly, please further justify use of IC10, rather than the more standard IC50, or IC90. I understand that this was chosen because IC10 shows higher heritability - again, a clearer explanation of heritability is required in the introduction/results.**

Thank you for the comment. We have explained how heritability was calculated in more detail and made changes to the manuscript to describe our heritability calculations (see our response in Comment #1 under Editor’s Comments to Author).

EC_10_, EC_50_, and EC_90_ values were all calculated. However, we are interested in the genes involved in anthelmintic resistance. Thus, we wanted to follow the phenotypic responses that are most heritable. EC_10_ is the effective concentration where the phenotypic responses of the six strains displayed the most heritable responses. This observation is also true across most species (from yeast to human cells) where quantitative phenotype data are gathered. S1 Fig shows the relationship between the calculated EC_10_ estimates and the doses with the most heritable responses. A detailed description of S1 Fig. can be found in our response to Comment #18.

We also know that EC_50_ is a noisier metric, especially when slope estimates (*b*) are steep which is observed in several of the anthelmintics highlighted in the manuscript. We hypothesize that the anthelmintic dosage closest to EC_10_ is more heritable than EC_50_ due to less variation within strains at the EC_10_ dosage.

**#5. Note that Rev 2 suggested replication of these results using an alternative approach to measuring resistance would improve confidence in the results. While valuable, this will not be required in the revision.**

Thank you for the suggestion. Although we appreciate the suggestion to perform additional assays, the presented data were collected from a large assay with high levels of replication, including a total of 48,343 replicate wells with up to 30 nematodes per well. Performing an additional assay at a similar scale would be a substantial undertaking. However, we have clarified why we have used body length as a metric of development (see our responses to Comments #2, #3, and #9) and addressed the misunderstanding about how the data were cleaned and filtered (see our responses to Comments #15, #46, and #47). We hope that our revisions and clarification satisfy Reviewer #2’s concerns.

**Part I - Summary**

**Reviewer #1:**

**#6. In this study, Dr. Shaver and colleagues authors used a panel of 6 genetically diverse C. elegans strains to perform dose-response analysis across 26 anthelmintic drugs, spanning 10 drug classes. They observed significant variation in anthelmintic responses (using microscopy-based high-throughput phenotyping assay for developmental delay in worms) of their C. elegans strains across drug classes, especially for the macrocyclic lactones and nicotinic acetylcholine receptor agonists. Finally, they quantified the heritability of the response to drugs. With this study, authors can prioritize drugs and populations of C.elegans to use for GWAS, in order to decipher the genetic basis and genes involved in drug resistance for multiple anthelmintics. Indeed, using genetically diverse populations is a key to identifying the right range of drug susceptibility and increasing the likelihood of identifying orthologous genes and mechanisms linked to drug resistance between C. elegans and parasitic nematodes.**

Thank you for the great summary.

**#7. Overall, the design of the study presented is very neat, with the appropriate controls and replications performed for each experiment, and an impressive number of individual tested. The analyses are also very robust, using the appropriate statistical tools and very well explained in the Materials & Methods section. Even if the manuscript is quite dense, it is well written and easy to understand, with great quality figures which are easily readable. One excellent point is that the authors have shared their datasets (Zenodo) and codes (Github), with is crucial in terms of rigor and reproducibility.**

Thank you for the great summary.

**#8. I have only a few minor comments and questions on the text (please see below for the details).**

Thank you for the critiques and feedback. We have done our best to respond to all of your

concerns.

**Reviewer #2:**

**#9. This paper addresses the important question of anthelmintic resistance using an elegant experimental approach that delineates the susceptibility of genetically diverse strains of C. elegans to gain insight into molecular determinants. The screen of six strains has been automated using a developmental assay, although for the sake of this relatively limited analysis it is not entirely clear what advantage that poses. This assay uses decrease in body length as a proxy for impaired development and anthelmintic effect. However, some of the compounds used will cause hypercontraction, or relaxation of the body wall muscle and this may be a confound for using body length as a metric. Furthermore, the automated approach is rather confounded by the effect of 48 hour exposure to some of the anthelmintics which may well be lethal and over this prolonged period would lead to the worms disintegrating and not amenable to a body length measurement. Indeed, this would seem to be the case and necessitated the use of filtering of the data set as indicated in the methods. The output of this study is the ability to identify drugs to prioritize for genome wide studies which ultimately will enable the identification of novel anthelmintic resistance genes. Overall the paper is well written and the work is carefully controlled.**

Thank you for this critique and the opportunity to explain our methods. To retain clarity, your comments and our responses are broken into several sections below:

A) Benefit of six strains in an automated assay:

The six strains used in this assay were chosen because they are a part of the CeNDR divergent strain set (<https://www.elegansvariation.org/strains/catalog>). These six strains represent 74% of the variation across the *C. elegans* species. Additionally, these six strains can be used to determine broad-sense heritability after collecting repeated independent measures of a phenotype such as body length used here. We have added a sentence in the *Methods* section *C. elegans* *strain selection and maintenance*:

“These six strains represent 74% of the variation across the *C. elegans* species”.

Also, see our responses regarding this topic in Comments #14 and #37.

The benefit of the automated assay is that we can assess high numbers of individuals to a variety of drug conditions in a high-throughput and rapid systematic fashion. Methods of this throughput are currently not possible in parasitic helminths. Thus, we can assess responses across genetically diverse *C. elegans* to any soluble compound of interest. We have the following statements in the manuscript to help clarify these points:

*Introduction*: “Lastly, because of the tractability of *C. elegans* and established high-throughput assays (HTA), we can measure *C. elegans* responses to any soluble compound [17].”

*Introduction*: “We measured nematode development after drug exposure for six genetically diverse *C. elegans* strains using an established high-throughput phenotyping assay [17]. We assayed the strains with high levels of replication, collecting a total of 48,343 replicate anthelmintic responses across genetically diverse *C. elegans* strains, a throughput not possible using parasites.”

*Results and Discussion*: “Our assays measured *C. elegans* developmental delay in the presence of anthelmintics across multiple concentrations of each drug. This study yielded several major findings.…

Second, the large-scale HTA provided quantitative data with the required statistical power and sample sizes needed to effectively measure anthelmintic responses.”

Lastly, the *Methods* section “*High-throughput anthelmintic dose-response assay*” describes this assay in detail.

B) Body length as the metric for development:

We acknowledge that there are many ways to assay anthelmintic response

beyond body length. We use body length as it is a reliable quantitative phenotypic metric for developmental rate. Our lab has used high-throughput assays and developed tools to measure body length as a trait to identify genes involved in anthelmintic resistance [[3–14]](https://paperpile.com/c/8HQFcn/gkcH+Dvaa+ShSr+riR4+LltC+4RNB4+fqvX+mB7u+Ovpj+qhVt+gReI+oxim).

We acknowledge that our findings and conclusions are based on using the metric of body length and have the following sentence in our *Results and Discussion*:

“Although little to no responses (*i.e.*, nematode development defects) were observed for five drugs using our assays, it is possible assays measuring different fitness traits could elicit anthelmintic effects.”

C) Hypercontraction:

Although BZs and NAChR agonists, for example, do cause muscle contractions,

this effect does not hinder our ability to adequately measure body length. After *C. elegans* have been exposed to anthelmintics for 48 hours, each well is treated with 50 mM sodium azide for 10 minutes to paralyze and strengthen nematodes prior to imaging.

We describe the details of this assay in the *Methods* under the subheading “*High-throughput anthelmintic dose-response assay*” and state:

“After 48 hours of incubation in the presence of food and anthelmintic, the 96-well microplates were removed from the incubator and treated with 50 mM sodium azide in M9 for 10 minutes to paralyze and straighten nematodes. Images of nematodes in the microplates were immediately captured using a Molecular Devices ImageXpress Nano microscope (Molecular Devices, San Jose, CA) using a 2X objective.”

D) 48-hour assay:

We explain our reason for using a 48-hour assay above in Comment #2 in the section “Editor’s comments to the author.” We hope this explanation helps clarify our assay choice. In short, we can measure the developmental delay from the L1 to the L4 larval stage and measure body length variation in a single generation.

E) Data filtering described in the methods:

Thank you for the opportunity to clarify our data cleaning and processing steps. We have clarified our cleaning steps in detail under Comment #46.

**Reviewer #3:**

**#10. The authors provide interesting and detailed analyses of five C. elegans natural isolates and the canonical wild type in their response to an impressive number (26) anthelmintic drugs. The study represents a lot of work and contains a lot of important information. However, I recommend that clarity be added to many of the sections to make the story easier to absorb for those who are not experts in population genetics. Detailed suggestions for improvement are listed below.**

**#10A. Abstract: It is not clear to me how the first point in the abstract (isolates respond differently to 2 of the 3 compounds tested) is different from the second point in the abstract (isolates respond differently to the compounds tested).**

Thank you for the opportunity to clarify these points. We have combined both statements in the abstract to read as follows:

“First, we found that *C. elegans* strains displayed similar anthelmintic responses within drug classes and significant variation across drug classes.”

**#10B. Abstract: The authors write, ‘Third, we quantified the heritability of responses to each anthelmintic and observed a significant correlation between exposure closest to the EC10 and the exposure that exhibited the most heritable responses.’. The exact meaning of ‘heritaility of responses’ is obscure to me. Is this a term common amoung population geneticists? Are the authors suggesting that the variation in the measured responses may not be heritable in the different isolates, and when tested in subsequent generations, they found that it was? Please clarify.**

Thank you for the opportunity to clarify these points about heritability. We have better-defined heritability and explained our methods. A description of our changes could be found in our responses to Comments #1, #18, #22, and #34. We hope these changes resolve any confusion around heritability.

**#10C. Abstract: The authors write, ‘Because genetically diverse strains displayed differential susceptibilities within and across anthelmintics, we show that C. elegans is a useful model for screening potential nematicides.’ It is not clear whether the authors mean that C. elegans is a useful pre-screening model to determine whether a candidate molecule is effective in all isolates, given their variable response, or whether they mean it is useful in some other way. Please clarify. I hope I will understand these points once I read the body of the manuscript, but the abstract needs clarity to be understood on its own.**

We apologize for the confusion. We have updated the quoted sentence in the abstract to clarify our intent. The sentence now reads:

“Because genetically diverse strains displayed differential susceptibilities within and across anthelmintics, we show that *C. elegans* is a useful model for screening potential nematicides before applications to helminths.”

**#11. Intro: Lines 107 and 108 (and 127-128): Reads as if WGS and AR variants from CeNDR are needed to identify orthologs between elegans and parasites. Certainly, identifying orthologs (best reciprocal matches by blastp for example) can be done without CeNDR. Perhaps the authors mean something else (e.g. within a paralogous families in different species, it is often difficult to determine which single genes are the true functional ortholog of one another by sequence alone)? Please clarify.**

Thank you for the opportunity to clarify. Reviewer #3 is referencing the following sentence: “Whole-genome sequence data and identified AR variants are available for CeNDR strains and can be queried to identify orthologous genes between *C. elegans* and parasites [[15]](https://paperpile.com/c/8HQFcn/jZATQ).”

We acknowledge that this wording is confusing, so we have altered it in the *Introduction*:

“Additionally, the natural genetic variation across the *C. elegans* species is accessible and continuously archived in the *C. elegans* Natural Diversity Resource (CeNDR), which has facilitated the characterization of natural responses to anthelmintic drugs [7,12,16]. Whole-genome sequence data and identified AR variants are available for all CeNDR strains, so orthologous genes between *C. elegans* and parasites can be queried to determine if *C. elegans* might be a good model for parasitic helminths [9].”

**#12. Line 111. Suggestion: ‘…have repeatedly proven to translate across parasitic nematode species (refs).’**

Thank you for the suggestion. We have accepted it, and the sentence now reads:

“Thus, the genetic diversity of *C. elegans* can enable the discovery of the molecular targets of anthelmintics and has repeatedly proven to translate across parasitic nematode species.”

**#13. Line 124. Can the authors expand a little on what they mean by ‘calculate the contributions of genetics’? Naively, I would assume that statistical variation within a given strain aside, that all differences in the response of strains to a drug is due to genetics. What else is there?**

What we mean by ‘calculate the contributions of genetics’ is measuring the heritability (*H_2_* and *h_2_*) of anthelmintic responses for the six strains. Some differences in response to a drug are environmental (V_E_), so heritability helps us determine the amount of that contribution.

**#14. Line 138. Explain how these 6 lines in particular were chosen.**

Thank you for the comment. Our response is below. We have also addressed this topic in

Comments #9 and #37.

The six strains used in this assay were chosen because they are a part of the CeNDR divergent strain set (<https://www.elegansvariation.org/strains/catalog>). These six strains contain 74% of the variation across the *C. elegans* species. Additionally, these six strains can be used to determine broad-sense heritability after collecting repeated independent measures of a phenotype such as body length used here. We have added a sentence in the Methods section *C. elegans strain selection and maintenance*:

“These six strains represent 74% of the variation across the *C. elegans* species”.

**#15. Line 139. Briefly, what does ‘cleaning data’ mean?**

Thank you for the opportunity to clarify what we mean by cleaning data.

We have two detailed sections in the Methods titled *Data cleaning* and *Small object removal* that explain all our data processing steps. Anytime ‘data cleaning’ is mentioned in the manuscript, we have inserted a note (*i.e.*, “(see *Data Cleaning*)” or “(see *Small object removal*)”) to help guide readers to the appropriate sections of the manuscript to understand these methods. Both the *Data cleaning* and *Small object removal* explain steps taken to ensure that we analyzed robust data. See Comment #46 for details.

**#16. Figure 1. More explanation in the legend is needed to explain step C. For example, what is OD30?**

Thank you for the suggestion. We have added more detail to the caption for Figure 1C. It now reads:

“**(C)** After 24 hours, a serial dilution containing eight concentrations for each anthelmintic was created. The serial dilution was added to an aliquot of *E. coli*. This mixture was fed to the nematodes.”

Additionally, we have the following sentence at the end of the Figure 1 caption to guide readers to the methods for thorough details about the steps in Figure 1:

“Detailed descriptions of all steps can be found in *Methods*.”

**#17. Line 163. I do not understand the concept of EC heritability. Please explain.**

Thank you for the opportunity to clarify. First, we calculated EC_10_ estimates using the *R/drc* package using a log-logistic model as described in the *Methods*:

“Dose-response model estimates and statistics have been modified from previous work [25]. We estimated overall and strain-specific dose-response models for each anthelmintic by fitting a log-logistic regression model using *R/drc* (Version 3.0.1) [71]. The four-parameter log-logistic function, *LL.4*, fits the anthelmintic data best. The *LL.4* model was fit to each anthelmintic using the *drc::drm()* function, where the model specified the following parameters: *b*, the slope of the dose-response curve; *c*, the upper asymptote of the dose-response curve; *d*, the lower asymptote of the dose-response curve; and *e*, the effective dose [72]. Strain was specified as a covariate for parameters *b* and *e*, allowing us to estimate strain-specific dose-response slopes and effective doses. The lower asymptote, *d*, was specified at -600, the theoretical normalized length of animals at the first larval stage.

The *drc::ED()* function was used to extract strain-specific EC_10_ values and strain-specific slope values [71]. We quantified the relative susceptibilities of each strain pair for each compound based on their estimated EC_10_ values using the *drc::EDcomp()* function, which used an approximate *F*-test to determine whether the variances (represented by delta-specified confidence intervals) calculated for each strain-specific dose-response model’s *e* parameter estimates were significantly different. We quantified the relative slope steepness of dose-response models estimated for each strain within each compound using the *drc::compParm()* function, which used a *z*-test to compare the means of each *b* parameter estimate. Results shown were filtered to comparisons against N2 dose-response parameters (**Fig 8**). Significantly different estimates in both cases were determined by correcting to a family-wise type I error rate of 0.05 using a Bonferroni correction. To determine whether strains were significantly more resistant or susceptible to more anthelmintics or anthelmintic classes by chance, we conducted 1000 Fisher exact tests using the *fisher.test()* function with 2000 Monte Carlo simulations.”

Using EC_10_ we then calculated how heritable anthelmintic responses were for each strain at each concentration of each drug. We have expanded our *Methods* section on heritability to now read:

**“Broad-sense and narrow-sense heritability calculations**

Phenotypic variance can be split into two parts, genetic variance (*V_G_*) and residual variance (*V_E_*). To determine the genetic contributions to phenotypic variance, we measured broad-sense (*H^2^*) and narrow-sense heritability (*h^2^*). Broad-sense heritability (*H^2^*) is the total fraction of phenotypic variance explained by genetic variation in a population [7]. Here, *H^2^* was calculated using the equation, *H^2^ = V_G_ / (V_G_+V_E_)*, where we extracted the among strain variance (*V_G_*) and residual variance (*V_E_*). Broad-sense heritability was estimated (*H^2^*) using in the R statistical environment (version 4.0.4) using the R package *lme4* (v1.1-27.1) to fit a linear mixed-effects model to the normalized median animal length data using strain as a random effect [25]. Genetic variance (*V_G_*) can be partitioned into additive (*V_A_*) and non-additive (*V_NA_*) variance components. Additive genetic variance (*V_A_*) is the amount of genetic variance explained by genotyped variants in a specific population. Narrow-sense heritability (*h^2^*) is the fraction of phenotypic variation explained by additive genetic variation in a population [7]. Narrow-sense heritability (*h^2^*) measures the additive genetic variance (*V_A_*) over the total phenotypic variance, *h^2^ = V_A_ / V_P_*. To calculate *h^2^*, we first generated a strain matrix using the strain *genomatrix profile* on NemaScan (https://github.com/AndersenLab/NemaScan), a genome-wide association (GWA) mapping and simulation pipeline [73]. NemaScan uses the GCTA software suite to calculate genomic relationship matrices [74,75] and used the hard-filtered variant call format (VCF) file ([WI.20220216.hard-filter.vcf.gz](https://storage.googleapis.com/caendr-site-public-bucket/dataset_release/c_elegans/20220216/variation/WI.20220216.hard-filter.vcf.gz)) generated in the 20220216 CeNDR release (<https://www.elegansvariation.org/data/release/latest>). The VCF used includes high-quality variants from 550 *C. elegans* isotypes, genetically distinct strains, after quality control filters. We then calculated *h^2^* using the *sommer* (v4.1.3) R package by calculating the variance-covariance matrix (*M_A_*) from the strain matrix using the *sommer::A.mat* function [25]. We estimated *V_A_* using the linear mixed-effects model function *sommer::mmer* using strain as a random effect and *M_A_* as the covariance matrix. We then estimated *h^2^* and its standard error using the *sommer::vpredict* function (**S16 Fig**). Lastly, to identify the amount of additive genetic variance contributing to *h2*, a correlation between the *h2* of the exposure closest to the estimated EC_10_ and the drug doses that exhibited the maximum *h2* for each anthelmintic with definitive EC_10_ estimates was performed (**S1 Fig**).”

**#18. S1 Fig. EC10. I do not understand the math. Someone else familiar with this will have to critique.**

We wanted to show how closely the exposures that exhibited the greatest narrow-sense heritability aligned with EC10 estimates for each anthelmintic. The narrow-sense heritability is between the exposure closest to the estimated EC10 and the exposures that exhibit the maximum narrow-sense heritability for each of the anthelmintics with definitive EC10 estimates were correlated. The correlation shows an extremely strong relationship between exposures that estimate the EC10 for each anthelmintic and the exposures that yielded the greatest narrow-sense heritability.

To help clarify this point, we have made the following edits:

In the *Methods* section *Broad-sense and narrow-sense heritability calculations,* we have added the following statement to discuss the correlation in S1 Fig:

“Lastly, to identify the amount of additive genetic variance contributing to *h2*, a correlation between the *h2* of the exposure closest to the estimated EC_10_ and the drug doses that exhibited the maximum *h2* for each anthelmintic with definitive EC_10_ estimates was performed (**S1 Fig, S6 Table**).”

All analysis steps can also be found in our R script on GitHub: <https://github.com/AndersenLab/anthelmintic_dose_responses_manuscript>

We have also added Supplementary Table 6 that summarizes the output values from calculating the heritability closest to EC_10_ for each anthelmintic. The table includes: the top heritable dose, the closest dose that shows an EC_10_ response, *H2* EC_10_, and *h2* EC_10_.

We also know that EC_50_ is a noisier metric, especially when slope estimates (*b*) are steep which is observed in several of the anthelmintics highlighted in the manuscript. We hypothesize that the anthelmintic dosage closest to EC_10_ is more heritable than EC_50_ due to less variation within strains at the EC_10_ dosage.

**#19. Line 170-172. It would be good to have an easily interpretable figure that shows the 44 instances across 22 compounds where one isolate behaves differently than N2.**

Thank you for the comment. Figure 8 shows how all six strains respond to all compounds measured. We represent N2 as an orange dotted line in Figures 8A and 8B. We felt that this comparison was important because N2 is the most widely used *C. elegans* strain and would highlight how natural genetic variation among the other five strains varies in comparison to N2. We describe that this figure shows responses compared to the N2 strain in the Figure 8 caption.

We also have included Figures S14 and S15 that show responses compared to N2 without the removal of MY16 for the BZs and DL238 for Milbemycin.

We hope this change addresses this concern.

**#20. Line 180. Need a display item to support claims.**

We believe the reviewer is referring to the following sentence: “Dose-response relationships described how different strains were affected at varying levels of anthelmintic exposure providing insights into how genetic differences impact anthelmintic susceptibility.”

We believe the combination of all dose-response curves, EC_10_ plots, slope plots, and heritability plots supports this claim. We have assayed six genetically diverse strains across 26 anthelmintics where we see variation in response across anthelmintics. These data show that strains are affected at varying levels of anthelmintic exposure showing that the genetic differences within these strains impact their anthelmintic response (*i.e.*, resistance or susceptibility).

We hope that we are not misinterpreting the Reviewer’s comment.

**#21. The data for the opening salvos of the paper (lines 167-191) are buried in difficult-to-absorb-quickly supplementary tables. Please have sympathy for the reader. Make it easy on us.**

We apologize that accessing and digesting the supplementary tables was difficult. To be as transparent and reproducible as possible, we wanted to provide the readers with the full slope (*b*) and effective concentration (*e*) outputs from the log-logistic model used. Therefore, we want to provide the full output table to readers. To help make these data digestible, we have provided the following figures: 3A, 3B, 5A, 5B, 7A, 7B, 8, S2, S14, and S15. We hope these figures help digest the slope (*b*) and effective concentration (*e*) results. Otherwise, we are happy to take specific suggestions to make the content easier to understand.

**#22. Give the reader some sense of what is meant by broad-sense and narrow-sense heritability without having to go the methods. These terms likely mean something very important to population geneticists, but mean nothing to the general reader of PLoS Pathogens. Even when I go to the methods (Lines 685-690), its all math talk without providing any intuitive meaning.**

Thank you for the opportunity to provide clarification on this topic. We have provided clarification about the definition of heritability and our methods used in the *Abstract*, *Introduction*, and *Methods*. Changes regarding this topic are detailed below.

We did add a brief definition of heritability in the *Abstract* that reads as follows:

“Third, we quantified the levels of anthelmintic response variation caused by genetic differences among individuals (heritability) to each drug and observed a significant correlation between exposure closest to the EC_10_ and the exposure that exhibited the most heritable responses.”

We added a sentence to define heritability in the *Introduction* that reads as follows:

“Over-reliance and inappropriate use of these anthelmintics have placed strong selective pressures on parasites and caused the evolution of anthelmintic resistance (AR) in every drug class [3]. In many cases, AR is highly heritable, which suggests that genetics plays an important role in the evolution of resistant nematodes [5,6]. Therefore, calculating the heritability of AR allows us to estimate the fraction of phenotypic variation in anthelmintic responses that can be explained by genetic variation in a population [7]. The narrow-sense heritability is a measure of the genetic component that is contributed by alleles that act additively so it can be used to calculate how much AR across a population will increase when mating resistant parents [6]. Because AR is highly heritable, we can identify the genes involved in AR and model resistance over time. These measures are critical because AR causes the drug to become ineffective [4]. With this knowledge, we can responsibly apply drugs and implement treatment strategies to reduce our global infection rate and burden of parasitic nematodes.”

We think the best place to have detailed definitions of both broad-sense and narrow-sense heritability belong in the *Methods*. Details about heritability have been added to the *Methods* section *Broad-sense and narrow-sense heritability calculations* which reads as:

**“Broad-sense and narrow-sense heritability calculations**

Phenotypic variance can be split into two parts, genetic variance (*V_G_*) and residual variance (*V_E_*). To determine the genetic contributions to phenotypic variance, we measured broad-sense (*H^2^*) and narrow-sense heritability (*h^2^*). Broad-sense heritability (*H^2^*) is the total fraction of phenotypic variance explained by genetic variation in a population [7]. Here, *H^2^* was calculated using the equation, *H^2^ = V_G_ / (V_G_+V_E_)*, where we extracted the among strain variance (*V_G_*) and residual variance (*V_E_*). Broad-sense heritability was estimated (*H^2^*) using in the R statistical environment (version 4.0.4) using the R package *lme4* (v1.1-27.1), to fit a linear mixed-effects model to the normalized median animal length data using strain as a random effect [25]. Genetic variance (*V_G_*) can be partitioned into additive (*V_A_*) and non-additive (*V_NA_*) variance components. Additive genetic variance (*V_A_*) is the amount of genetic variance explained by genotype variants that differ in a specific population. Narrow-sense heritability (*h^2^*) is the fraction of phenotypic variation explained by additive genetic variation in a population [7]. Narrow-sense heritability (*h^2^*) measures the additive genetic variance (*V_A_*) over the total phenotypic variance, *h^2^ = V_A_ / V_P_*. To calculate *h^2^*, we first generated a strain matrix using the strain *genomatrix profile* on NemaScan (https://github.com/AndersenLab/NemaScan), a genome-wide association (GWA) mapping and simulation pipeline [73]. NemaScan uses the GCTA software suite to calculate genomic relationship matrices [74,75] and used the hard-filtered variant call format (VCF) file ([WI.20220216.hard-filter.vcf.gz](https://storage.googleapis.com/caendr-site-public-bucket/dataset_release/c_elegans/20220216/variation/WI.20220216.hard-filter.vcf.gz)) generated in the 20220216 CeNDR release (<https://www.elegansvariation.org/data/release/latest>). The VCF used includes high-quality variants from 550 *C. elegans* isotypes, genetically distinct strains, after quality control filters. We then calculated *h^2^* using the *sommer* (v4.1.3) R package by calculating the variance-covariance matrix (*M_A_*) from the strain matrix using the *sommer::A.mat* function [25]. We estimated *V_A_* using the linear mixed-effects model function *sommer::mmer* using strain as a random effect and *M_A_* as the covariance matrix. We then estimated *h^2^* and its standard error using the *sommer::vpredict* function (**S16 Fig**). Lastly, to identify the amount of additive genetic variance contributing to *h2*, a correlation between the *h2* of the exposure closest to the estimated EC_10_ and the drug doses that exhibited the maximum *h2* for each anthelmintic with definitive EC_10_ estimates was performed (**S1 Fig. S6 Table**).”

We hope these additions help.

**#23. Lines 224-226: ‘ This heritable response indicated that genetic differences among the six strains underlie the variation in albendazole responses.’ Does one really need to do broad-sense and narrow-sense heritability calculations to come to this conclusion? The null hypothesis makes no sense- the differences in the response to the strains are not due to genetic differences’. It must be due to genetic differences, unless I am missing something.**

Thank you for the comment, and we apologize for the confusion. We make this statement because albendazole has the highest heritable responses out of the five benzimidazoles assayed. Without heritability calculations, you would indeed see that the six strains have variable responses to all five benzimidazoles. However, you would not know how heritable the responses assayed are because phenotypic variation can also be caused by environmental factors (*e.g.*, experimental noise). By calculating the heritability of the phenotypic responses to each anthelmintic, we start to disentangle the complexity of these traits. Heritability helps create inferences about how AR is selected in natural populations.

**#24. Line 399. Why hypothesized?**

The reviewer is referring to the following sentence: “Drugs outside of the three major anthelmintic classes are valuable because they have different hypothesized targets and mechanisms of resistance that could be effective against nematodes resistant to other drug classes.”

We use the word hypothesized to describe the targets and mechanisms of resistance for anthelmintic classes outside of BZs, MLs, and NAChR agonists because they are not well understood. Modes of action (MoA) and genes have been implicated in the resistance of these anthelmintics, but much work is left to be done to understand the mechanisms of resistance. Therefore, we used the word hypothesized.

**#25. Line 479. Can the authors explain how growth trends ‘can be used to assess how other nematodes might respond to the tested anthelmintic drugs’?**

The reviewer is referring to the following sentence: “Growth trends yielded information that can be used to assess how other nematodes might respond to the tested anthelmintic drugs.”

First, we have changed this sentence to now read: “Dose-response trends yielded information that can be used to assess how other nematodes might respond to the tested anthelmintic drugs.” In Comment #43 a Reviewer had a concern with using the term ‘growth’, so we have edited this sentence.

Because compound evaluation is difficult to perform in parasitic helminths due to access to relevant life cycle stages, host-dependent life cycles, high laboratory costs, and limited molecular tool kits, *C. elegans* can be used and has been used as a model to test anthelmintic compounds. Here, we suggest that *C. elegans* can be used to assess anthelmintic responses, and those findings can provide a foundational expectation of how effective an anthelmintic can be.

The following sentences in the *Introduction* provide background on these points:

“It is difficult to accurately test AR in genetically diverse parasitic nematodes because of multiple factors: a lack of access to relevant life cycle stages, lack of global sample collections, host-dependent and cost-intensive laboratory life cycles, complex or non-existent *in vitro* culture systems, and a limited molecular toolkit [8].

With its ease of growth, genetic tractability, and ample molecular toolkit, the roundworm *C. elegans* is our most useful model to study AR. To date, *C. elegans* has contributed to the identification and characterization of mechanisms of resistance of all major anthelmintic drug classes [9–15].”

**#26. Lines 480-482. I don’t think a summary of the methodology used to be a major finding, as intended on line 477.**

Thank you for the suggestion. We have split the sentence of concern. The sentences now read:

“Our assays measured *C. elegans* growth rates in the presence of anthelmintics across multiple concentrations of each drug. This study yielded several major findings.”

**#27. Lines 495-496. Can the authors provide proof-of-principle from their data of how using multiple isolates might help yield a candidate anthelmintic that could not be identified by using only N2?**

The reviewer is referring to the following sentence: “We suggest that genetically diverse *C. elegans* strains should be deployed to aid high-throughput anthelmintic screening efforts to identify effective anthelmintics and estimated effective concentrations to use when testing in parasitic nematodes.”

First, we explain the value of using genetically diverse *C. elegans* strains to identify the mechanisms of resistance in our *Introduction*.

In the second paragraph of the *Introduction,* we mention the following:

“Most of what we know about mechanisms of resistance comes from studies of a single strain within a single species, the laboratory-adapted strain of *Caenorhabditis elegans* called N2. However, a single genetic background, whether in a free-living or parasitic species, cannot capture the enormous diversity present in the entire species, nor can it predict how natural populations of parasitic nematodes will respond to a drug [[16]](https://paperpile.com/c/8HQFcn/LNePN). In aggregate, single strains from many species might capture phylum-level variation, which strengthens the opportunity to identify genes involved in mechanisms of resistance.”

In the third paragraph of the *Introduction*, we show proof-of-principle support of how natural isolates have aided in characterizing the natural responses to anthelmintic drugs:

“With its ease of growth, genetic tractability, and ample molecular toolkit, the roundworm *C. elegans* is our most useful model to study AR. To date, *C. elegans* has contributed to the identification and characterization of mechanisms of resistance of all major anthelmintic drug classes [[15,17–22]](https://paperpile.com/c/8HQFcn/jZATQ+YE36Q+2o8aZ+84hRL+OMx9S+vTx5s+MZxnm). Additionally, the natural genetic variation across the *C. elegans* species is accessible and continuously archived in the *C. elegans* Natural Diversity Resource (CeNDR), which has facilitated the characterization of natural responses to anthelmintic drugs [[8,19,23]](https://paperpile.com/c/8HQFcn/4RNB4+84hRL+s51wu).”

In the results, we present examples of how variation among these six strains provides value to understand resistance mechanisms. For example, in the benzimidazoles (Figures 2 and 3), it is clear that strain MY16 (gray) is highly resistant to all five benzimidazoles, whereas N2 is more sensitive. This finding suggests that strain MY16 carries some genetic variant that allows it to be resistant in this drug class. Indeed, we know that the MY16 strain has a non-synonymous variant in the beta-tubulin gene *ben-1*, causing an amino acid change (A185P) [[24]](https://paperpile.com/c/8HQFcn/Qc0rz) and a presumptive reduction in *ben-1* function. This example shows how genetic variation in a natural isolate can aid in the elucidation of resistance mechanisms that may not be found in N2 alone. It is important to note that Sydney Brenner could have been sent a different *C. elegans* isolate (*e.g.*, MY16) that is naturally resistant to BZ compounds and our understanding of BZ resistance would have been significantly delayed. For other anthelmintics, we show that responses vary. The N2 strain could be more resistant than the average strain hindering the ability to do genetic screens or other molecular assays to elucidate resistance mechanisms. We have added these points to the *Results and Discussion* as shown below:

“Because most studies in *C. elegans* AR have been conducted using the laboratory reference strain, N2, or mutant strains in the N2 genetic background, these results emphasize the importance of using genetically diverse individuals to understand drug responses. Furthermore, the observed frequency of strains with significantly greater anthelmintic sensitivity than the N2 strain was different than what is expected under the null expectation (see *Methods*; Fisher exact test; *p* < 0.05), which suggests that diverse *C. elegans* strains are not equally likely to be susceptible or resistant with respect to the commonly used N2 reference strain.”...

“These results support the previous findings that *ben-1* is not the only gene involved in BZs resistance and that diverse *C. elegans* strains vary across a spectrum of BZ responses [20,36,38]. The strain MY16 is a striking example of how natural BZ resistance alleles can protect nematodes from BZ treatment. In the context of natural parasitic nematode populations, it is easy to imagine how such beneficial alleles could spread rapidly and further exacerbate parasitic burdens. This example shows how genetic variation in a natural isolate can aid in the elucidation of resistance mechanisms that may not be found in N2 alone. It is important to note that Sydney Brenner could have been sent a different *C. elegans* isolate (*e.g.*, MY16) that is naturally resistant to BZs, and our understanding of BZ resistance would have been significantly delayed.”

**#28. After examining Figures 3a, 3b, 5a, 5b, 7a, and 7b, it is not clear that there are important/significant insights gained from the slope analyses that are not gained from EC10 analyses alone.**

Thank you for the comment. We agree that the trends observed in the EC_10_ plots (3a, 5a, and 7a) are similar to those observed in the slope plots (3b, 5b, and 7b). Although similar, they are not identical. In order to give the community the full results from the log-logistic model, we believe that both the EC_10_ and slope estimates are valuable. Historically, the veterinary parasitology literature reports effective concentration (EC) estimates along with slope estimates. Because we hope that this manuscript will be a resource to that community, we believe reporting both values will be useful to the reader. Lastly, the slope values yield information about the change in response per unit dose of the anthelmintic that EC_10_ does not provide.

**#29. Figure 8 is a really nice summary of data presented in 3a, 3b, 5a, 5b, 7a, and 7b, along with additional data. It begs the question of whether 3a, 3b, 5a, 5b, 7a, and 7b are needed at all?**

Thank you for the comment. Figure 8 is a summary of response of all strains across all anthelmintics with respect to N2 (the orange dashed line in Figure 8). Figure 8 displays the value of why natural variation is critical to consider when understanding anthelmintic responses, because one strain (N2) cannot provide all information about anthelmintic responses for a species. Additionally, Figure 8 shows if any of the five strains have significantly different responses to a drug compared to N2 (solid points) or not (faded points). Figure 8 is all in relation to N2.

The other figures (3, 5, and 7) give the reader the opportunity to see (A) how the six strains respond to each drug individually allowing for a better view of the trends identified, (B) provides the units of EC10 estimates for each anthelmintic, (C) provides the units of the slope for each anthelmintic, and (D) heritability for each concentration of each drug.

**Part II – Major Issues: Key Experiments Required for Acceptance**

**Reviewer #1:**

**#30. None**

**Reviewer #2:**

**#31. It would lend confidence to the work if some of the key findings were reproduced using another assay of anthelmintic susceptibility. The use of body length as a read out of the impact of the compounds on development and thus a measure of sensitivity has confounds as noted above. Moreover, the analysis required what appears to be extensive filtering of the data set.**

We appreciate your response. Because this study was an incredibly large set of assays with high levels of replication, including a total of 48,343 replicate wells with up to 30 nematodes per well, repeating a similar study would be an incredible undertaking. We have addressed concerns about using body length as a metric of development above (see Comments #2, #3, and #9). We have also addressed the misunderstanding about how the data were cleaned and filtered (see Comments #15 and #46).

**Reviewer #3:**

**#32. (No Response)**

**Part III – Minor Issues: Editorial and Data Presentation Modifications**

**Reviewer #1:**

**Results/Discussion:**

**#33. 162-163 - Do the authors have a hypothesis to explain why the EC10 estimates are more heritable than the EC50 estimates?**

We know that EC_50_ is a noisier metric, especially when slope estimates (*b*) are steep which is observed in several of the anthelmintics highlighted in the manuscript. We hypothesize that the anthelmintic dosage closest to EC_10_ is more heritable than EC_50_ due to less variation within strains at the EC_10_ dosage.

**#34. 298-299: This sentence seems a bit unclear: Why do the authors not provide the h2 value for ivermectin (only the H2 value is mentioned) and the H2 value for doramectin (only the h2 value is mentioned)?**

Thank you for pointing out this difficult-to-interpret section. The Reviewer is referring to the following sentences: “To quantify the degree of phenotypic variation attributable to segregating genetic differences among strains, we estimated the *H^2^* and *h^2^* of the phenotypic responses in all MLs (**Fig 5C**). We observed that *H^2^* ranged from 0.02 in 0.00533 µM ivermectin to 0.87 in 0.27 µM milbemycin, and *h^2^* ranged from 0.01 in 0.00105 µM doramectin to 0.73 in 0.27 µM milbemycin.”

These summary sentences aim to showcase the range of *H^2^* and *h^2^* obtained in each drug class and highlight the most and least heritable responses. For each drug class, we list the drug and concentration with the smallest *H^2,^* the drug and concentration with the largest *H^2^*, and the same for *h^2^*.

The reason ivermectin is mentioned when describing *H^2^* for the macrocyclic lactones (MLs) is because ivermectin at a concentration of 0.00533 µM had the smallest broad-sense heritability (*H^2^*) of 0.02. Conversely, milbemycin at a concentration of 0.27 µM had the highest broad-sense heritability (*H^2^*) of 0.87. Here, we only mention ivermectin and milbemycin because they are the low and high extremes for broad-sense heritability (*H^2^* ) in the MLs.

The reason doramectin is mentioned when describing *h^2^* for the macrocyclic lactones (MLs) is because doramectin at a concentration of 0.00105 µM had the smallest narrow-sense heritability (*h^2^*) of 0.01. Conversely, milbemycin at a concentration of 0.27 µM had the highest narrow-sense heritability (*h^2^*) of 0.73. Here, we only mention doramectin and milbemycin because they are the low and high extremes for narrow-sense heritability (*h^2^*) in the MLs.

Each sentence that delineates narrow-sense and broad-sense heritability by anthelmintic class states that we are reporting the ***range*** of heritability.

**Figures:**

**#35. Dose-Response curves Figures - legend: For each strain and each drug dose, how many worms have been treated? Adding N values to the legends of the dose-response curves would be useful.**

Thank you for the suggestion. After all *Data cleaning* steps (see *Methods*), we have 48,343 replicate anthelmintic responses across the six strains and anthelmintics. The minimum number of replicates (wells) needed to proceed with analysis per anthelmintic, condition, and strain is ten.

We added a sentence to the *Methods* section, “*Dose-response model estimation and statistics*” that reads:

“After *Data cleaning* and *Small object removal* steps, each anthelmintic dose contained a minimum of 10 replicates per strain, with a minimum of 30 nematodes, to proceed with dose-response model estimates and statistics.”

We hope that this addition clarifies the minimum number of nematodes needed per strain per anthelmintic condition to proceed with the analysis. We do not believe that adding *N* values to each dose for each strain and each anthelmintic would be easily interpretable.

**Tables:**

**#36. Table 1: Perhaps worth adding a column to the Table 1 mentioning the range of doses used for each drugs in this study.**

Thank you for the suggestion. Supplementary Table 5 includes full details about each

anthelmintic used. We previously reported the highest dose of each anthelmintic used in this table. We have now added columns with the full range of doses for each drug to Supplementary Table 5, as suggested.

**Materials & Methods:**

**#37. C. elegans strain selection and maintenance: What is the percentage of genetic divergence between the different stains chosen? Perhaps good to mention in this paragraph. This will clarify, for the reader, why these specific 6 C. elegans strains have been chosen, especially if they are very divergent genetically between each other.**

Thank you for the suggestion. We have added the following sentences to the *Materials and Methods* section *C. elegans strain selection and maintenance*:

“These six strains represent 74% of the variation across the *C. elegans* species”.

**#38. High-throughput anthelmintic dose-response assay:** **In this experimental design, the worms were exposed to various doses of drugs during 48h. Why has this specific treatment/incubation time have been chosen? Have the authors observed similar results with a shorter treatment time (24h)? Would it be possible to treat worms for 24h and then culture them for 24h without drug and assess their phenotypic response? I suspect it would be difficult, as these worms are tiny, but it would better mimic what could happen in-vivo with parasitic nematodes, when hosts are treated with anthelmintics. Perhaps good here to justify briefly why this specific exposure time was chosen.**

Thank you for the suggestion. This particular high-throughput phenotyping assay has been robustly tested in our lab and provided phenotyping results for several assays [[12,13]](https://paperpile.com/c/8HQFcn/qhVt+gReI). We used this assay because we could synchronize all strains at the L1 larval stage and expose nematodes to drugs over 48 hours to allow nematodes to develop to the L4 stage. This treatment allows us to collect data from various strains exposed to many anthelmintics under the same time frame and conditions. This time frame was selected so that we could follow the development of worms over several larval stages before nematodes become gravid, lay embryos, and proceed to the next generation.

Because a high-level of replication was performed in these dose-response assays, we are confident in our results measuring body length.

We thank Reviewer #1 for the experimental suggestion but believe that the earlier development assay would have confounding issues, such as carrying over residual anthelmintics at a low exposure after transfer at the 24-hour mark and adding additional transfer steps and potential loss of sample. Additionally, as mentioned in Comment #2 mimicking *in vivo* assays is difficult even within helminths so we believe that we should describe the assay that we have used in enough detail to be interpreted and reproduced.

**COMMENTS ON THE REFERENCES**

**Reviewer #2:**

**#39. None**

**Reviewer #2:**

**#40. Why would EC10 estimates be more heritable than EC50 estimates? A comment of this**

**would be helpful.**

Thank you for the comment. Please see our response to Comment #33.

**#41. Its not helpful having so much of the data as supplementary information.**

Thank you for the comment. In the main manuscript, we focus on the three major anthelmintic drug classes (BZs, MLs, and NAChR agonists) used in veterinary parasitology (Figures 3 - 7). Figure 8 gives an overview of *C. elegans r*esponses over all tested anthelmintics.

To facilitate the flow of content in the main body of the manuscript but also to provide researchers with findings across all 26 anthelmintics, we have put figures pertaining to additional anthelmintics (not in the three main classes) and data cleaning steps in the supplementary information. Additionally, although we distilled the content from the supplementary tables into figures in the main manuscript, we want to provide these data to the community to use for their own work. All figures and data files can be accessed on GitHub. We hope this organization will help most readers easily access the information.

**#42. Fig 8. How was relative potency calculated?**

Thank you for the opportunity to clarify what we mean by ‘relative potency.’ Relative potency is referring to the relative steepness of dose-response slope estimates compared to the N2 reference strain, analogously to our EC10 relative potency analysis.

We have explained the details of how potency was calculated in the *Methods* section *Dose-response model estimation and statistics*. We have the following statement:

“We quantified the relative slope steepness of dose-response models estimated for each strain within each compound using the *drc::compParm()* function, which used a *z*-test to compare the means of each *b* parameter estimate. Results shown were filtered to comparisons against N2 dose-response parameters (**Fig 8**). Significantly different estimates in both cases were determined by correcting to a family-wise type I error rate of 0.05 using a Bonferroni correction. To determine whether strains were significantly more resistant or susceptible to more anthelmintics or anthelmintic classes by chance, we conducted 1000 Fisher exact tests using the *fisher.test()* function with 2000 Monte Carlo simulations.”

Calculations for relative potency and all statistics can be found in our R script ([master_anthDRmanuscript_analysis_script.R](https://github.com/AndersenLab/anthelmintic_dose_responses_manuscript/blob/main/scripts/master_anthDRmanuscript_analysis_script.R)) on GitHub: https://github.com/AndersenLab/anthelmintic_dose_responses_manuscript/tree/main/scripts

**#43. Line 458 – only one phenotype was measured, so this is a bit of an overstatement**

Reviewer #2 is referring to the following statement “Dose-response assessment across genetically diverse stains identifies five anthelmintics for which *C. elegans* had little to no phenotypic responses”.

We agree that we only use one trait in this manuscript. We state that we see little to no phenotypic responses for five of the drugs tested. At the end of the paragraph, we do state the following:

“Although little to no responses (*i.e.*, nematode growth defects) were observed for five drugs using our assays, it is possible assays measuring different fitness traits could elicit anthelmintic effects.”

We hope this statement satisfies the reviewer's concern.

**#44. Line 476- analysis did not measure growth rates- it only measured an end point**

Thank you for the comment. We have changed this sentence to now read:

“Our assays measured *C. elegans* developmental delay in the presence of anthelmintics across multiple concentrations of each drug.”

Additionally, we searched for any other mention of “growth rate” in the manuscript and altered our language to mention “developmental delay” instead.

**#45. Add information on the mode of action of the different compounds e.g., add to table 1**

Thank you for the suggestion. We have added a “Mode of Action (MoA)” column to Table 1 and

have listed details for anthelmintics with known MoA. We also updated the Table 1 caption to

Read:

“**Table 1.** Drug class, subclass, and drugs used in this study. Drug classes with defined

Mode of Action (MoA) are listed.”

A screenshot of the changes to Table 1 below:

**#46. Line 616- what is this 'debris'? dead worms? how can this be excluded if this is the**

**case?**

Reviewer #2 is referencing the following sentence: “Drugs with an abundance of objects

classified by the MDHD model across assays and concentrations likely contain debris.”

We apologize for the lack of clarity. Debris are either *E. coli* bacterial clumps, crystals from a solubility issue, or contaminants. The debris is not dead worms. We want to remove as much debris (*i.e.*, non-worm objects) as possible so as not to confound our worm length measurements in a well and create unwanted variation.

We have edited the referenced sentence for clarity. The sentence now reads:

“Drugs with an abundance of objects classified by the MDHD model across assays and concentrations likely contain debris (*i.e.*, bacterial clumps, contaminants, or drug crystals).”

**#47. line 624- 30 worms were the starting point but less than 3 in a well excluded? This suggests very large variation in the data**

Reviewer #2 is referring to the following sentence: “Given that our analysis relied on well median animal length measurements, we excluded wells with less than three animals to

reduce sampling error.”

Thank you for the comment. Although we titered wells to have approximately 30 embryos per well, we agree variation in animal counts exists. We have cleaning parameters in place to ensure that we do not include wells with fewer than three animals and more than forty animals to ensure that we can perform our larval stage modeling (CellProfiler) and statistics. The Methods *Data cleaning* section lays out the parameters the data must meet to ensure that we are using high-quality data in the analysis. We talk about well *N* variation in the following statement:

“We then filtered the data to wells containing between three and forty animals, under the null hypothesis that the number of animals is an approximation of the expected number of embryos originally titered into wells (approximately 30). Given that our analysis relied on well median animal length measurements, we excluded wells with fewer than three animals to reduce sampling error.”

Additionally, more than three animals are in each well, but particular parameters put in place in the *easyXpress* steps can reduce the total worms counted in the well *N*. Two steps in the *easyXpress* process can reduce animal *N*: (1) worms along the perimeter of a well are not counted due to variation in exposure around the edges of a well, and (2) any worms touching or overlapping are not counted to ensure greatest accuracy of the worm models.

These parameters and details are reported in a previous publication (Nyaanga J, Crombie TA, Widmayer SJ, Andersen EC (2021) easyXpress: An R package to analyze and visualize high-throughput *C. elegans* microscopy data generated using CellProfiler. PLoS ONE 16(8): e0252000. https://doi.org/10.1371/ journal.pone.0252000). We have cited this manuscript and stated the following:

“Next, a custom R package, *easyXpress* (Version 1.0), was then used to process animal measurements output from CellProfiler [17].”

**Reviewer #3:**

**#48. (No Response)**

**References cited in this letter:**

1.	[Yang J, Lee SH, Goddard ME, Visscher PM. GCTA: a tool for genomewide complex trait analysis. Am J Hum Genet. 2011;88: 76–82.](http://paperpile.com/b/8HQFcn/6Dug)

2.	[Jiang L, Zheng Z, Qi T, Kemper KE, Wray NR, Visscher PM, et al. A resource-efficient tool for mixed model association analysis of large-scale data. Nat Genet. 2019;51: 1749–1755.](http://paperpile.com/b/8HQFcn/eqk2)

3.	[Zdraljevic S, Strand C, Seidel HS, Cook DE, Doench JG, Andersen EC. Natural variation in a single amino acid substitution underlies physiological responses to topoisomerase II poisons. PLoS Genet. 2017;13: e1006891.](http://paperpile.com/b/8HQFcn/gkcH)

4.	[Zamanian M, Cook DE, Zdraljevic S, Brady SC, Lee D, Lee J, et al. Discovery of genomic intervals that underlie nematode responses to benzimidazoles. PLoS Negl Trop Dis. 2018;12: e0006368.](http://paperpile.com/b/8HQFcn/Dvaa)

5.	[Zdraljevic S, Fox BW, Strand C, Panda O, Tenjo FJ, Brady SC, et al. Natural variation in C. elegans arsenic toxicity is explained by differences in branched chain amino acid metabolism. Elife. 2019;8. doi:](http://paperpile.com/b/8HQFcn/ShSr)[10.7554/eLife.40260](http://dx.doi.org/10.7554/eLife.40260)

6.	[Gibson SB, Ness-Cohn E, Andersen EC. Benzimidazoles cause lethality by inhibiting the function of Caenorhabditis elegans neuronal beta-tubulin. bioRxiv. 2022. p. 2022.07.21.500991. doi:](http://paperpile.com/b/8HQFcn/riR4)[10.1101/2022.07.21.500991](http://dx.doi.org/10.1101/2022.07.21.500991)

7.	[Pallotto LM, Dilks CM, Park Y-J, Smit RB, Lu B, Gopalakrishnan C, et al. Interactions of C. elegans β-tubulins with the microtubule inhibitor albendazole. bioRxiv. 2022. p. 2022.02.27.482202. doi:](http://paperpile.com/b/8HQFcn/LltC)[10.1101/2022.02.27.482202](http://dx.doi.org/10.1101/2022.02.27.482202)

8.	[Evans KS, van Wijk MH, McGrath PT, Andersen EC, Sterken MG. From QTL to gene: C. elegans facilitates discoveries of the genetic mechanisms underlying natural variation. Trends Genet. 2021;37: 933–947.](http://paperpile.com/b/8HQFcn/4RNB4)

9.	[Evans KS, Wit J, Stevens L, Hahnel SR, Rodriguez B, Park G, et al. Two novel loci underlie natural differences in Caenorhabditis elegans macrocyclic lactone responses. bioRxiv. 2021. p. 2021.01.14.426644. doi:](http://paperpile.com/b/8HQFcn/fqvX)[10.1101/2021.01.14.426644](http://dx.doi.org/10.1101/2021.01.14.426644)

10.	[Evans KS, Wit J, Stevens L, Hahnel SR, Rodriguez B, Park G, et al. Two novel loci underlie natural differences in Caenorhabditis elegans abamectin responses. PLoS Pathog. 2021;17: e1009297.](http://paperpile.com/b/8HQFcn/mB7u)

11.	[Wit J, Rodriguez BC, Andersen EC. Natural variation in Caenorhabditis elegans responses to the anthelmintic emodepside. Int J Parasitol Drugs Drug Resist. 2021;16: 1–8.](http://paperpile.com/b/8HQFcn/Ovpj)

12.	[Widmayer SJ, Crombie TA, Nyaanga JN, Evans KS, Andersen EC. C. elegans toxicant responses vary among genetically diverse individuals. Toxicology. 2022; 153292.](http://paperpile.com/b/8HQFcn/qhVt)

13.	[Nyaanga J, Crombie TA, Widmayer SJ, Andersen EC. easyXpress: An R package to analyze and visualize high-throughput C. elegans microscopy data generated using CellProfiler. PLoS One. 2021;16: e0252000.](http://paperpile.com/b/8HQFcn/gReI)

14.	[Brady SC, Zdraljevic S, Bisaga KW, Tanny RE, Cook DE, Lee D, et al. A Novel Gene Underlies Bleomycin-Response Variation in Caenorhabditis elegans. Genetics. 2019;212: 1453–1468.](http://paperpile.com/b/8HQFcn/oxim)

15.	[Hahnel SR, Dilks CM, Heisler I, Andersen EC, Kulke D. Caenorhabditis elegans in anthelmintic research – Old model, new perspectives. International Journal for Parasitology: Drugs and Drug Resistance. 2020. pp. 237–248. doi:](http://paperpile.com/b/8HQFcn/jZATQ)[10.1016/j.ijpddr.2020.09.005](http://dx.doi.org/10.1016/j.ijpddr.2020.09.005)

16.	[Hahnel, Dilks, Heisler, Andersen. Caenorhabditis elegans in anthelmintic research–Old model, new perspectives. Int J Educ Vocat Guid. 2020. Available:](http://paperpile.com/b/8HQFcn/LNePN) <https://www.sciencedirect.com/science/article/pii/S2211320720300312>

17.	[Dilks CM, Hahnel SR, Sheng Q, Long L, McGrath PT, Andersen EC. Quantitative benzimidazole resistance and fitness effects of parasitic nematode beta-tubulin alleles. Int J Parasitol Drugs Drug Resist. 2020;14: 28–36.](http://paperpile.com/b/8HQFcn/YE36Q)

18.	[Dilks CM, Koury EJ, Buchanan CM, Andersen EC. Newly identified parasitic nematode beta-tubulin alleles confer resistance to benzimidazoles. Int J Parasitol Drugs Drug Resist. 2021;17: 168–175.](http://paperpile.com/b/8HQFcn/2o8aZ)

19.	[Wit J, Dilks CM, Andersen EC. Complementary Approaches with Free-living and Parasitic Nematodes to Understanding Anthelmintic Resistance. Trends Parasitol. 2021;37: 240–250.](http://paperpile.com/b/8HQFcn/84hRL)

20.	[Kwok TCY, Ricker N, Fraser R, Chan AW, Burns A, Stanley EF, et al. A small-molecule screen in C. elegans yields a new calcium channel antagonist. Nature. 2006;441: 91–95.](http://paperpile.com/b/8HQFcn/OMx9S)

21.	[Leung CK, Wang Y, Malany S, Deonarine A, Nguyen K, Vasile S, et al. An ultra high-throughput, whole-animal screen for small molecule modulators of a specific genetic pathway in Caenorhabditis elegans. PLoS One. 2013;8: e62166.](http://paperpile.com/b/8HQFcn/vTx5s)

22.	[Lemieux GA, Liu J, Mayer N, Bainton RJ, Ashrafi K, Werb Z. A whole-organism screen identifies new regulators of fat storage. Nat Chem Biol. 2011;7: 206–213.](http://paperpile.com/b/8HQFcn/MZxnm)

23.	[Cook DE, Zdraljevic S, Roberts JP, Andersen EC. CeNDR, the Caenorhabditis elegans natural diversity resource. Nucleic Acids Res. 2017;45: D650–D657.](http://paperpile.com/b/8HQFcn/s51wu)

24.	[Latheef SLJ. The use of natural genetic variation in Caenorhabditis elegans to identify novel polymorphisms that can confer benzimidazole resistance in nematodes. Graduate Studies; 2014. doi:](http://paperpile.com/b/8HQFcn/Qc0rz)[10.11575/PRISM/28412](http://dx.doi.org/10.11575/PRISM/28412)
